# Gene expression analyses of primary melanomas reveal CTHRC1 as an important player in melanoma progression

**DOI:** 10.18632/oncotarget.7604

**Published:** 2016-02-23

**Authors:** Johanna Eriksson, Vadim Le Joncour, Pirjo Nummela, Tiina Jahkola, Susanna Virolainen, Pirjo Laakkonen, Olli Saksela, Erkki Hölttä

**Affiliations:** ^1^ Department of Pathology, University of Helsinki, FI-00014 Helsinki, Finland; ^2^ University of Helsinki, Research Programs Unit, Translational Cancer Biology, Biomedicum Helsinki, FI-00014 Helsinki, Finland; ^3^ Department of Plastic Surgery, Helsinki University Central Hospital, FI-00029 Helsinki, Finland; ^4^ Department of Dermatology, Helsinki University Central Hospital, FI-00029 Helsinki, Finland

**Keywords:** melanoma, invasion/metastasis, CTHRC1, NFATC2, TGFβ

## Abstract

Melanoma is notorious for its high tendency to metastasize and its refractoriness to conventional treatments after metastasis, and the responses to most targeted therapies are short-lived. A better understanding of the molecular mechanisms behind melanoma development and progression is needed to develop more effective therapies and to identify new markers to predict disease behavior. Here, we compared the gene expression profiles of benign nevi, and non-metastatic and metastatic primary melanomas to identify any common changes in disease progression. We identified several genes associated with inflammation, angiogenesis, and extracellular matrix modification to be upregulated in metastatic melanomas. We selected one of these genes, collagen triple helix repeat containing 1 (*CTHRC1*), for detailed analysis, and found that CTHRC1 was expressed in both melanoma cells and the associated fibroblasts, as well as in the endothelium of tumor blood vessels. Knockdown of CTHRC1 expression by shRNAs in melanoma cells inhibited their migration in Transwell assays and their invasion in three-dimensional collagen and Matrigel matrices. We also elucidated the possible down-stream effectors of CTHRC1 by gene expression profiling of the CTHRC1-knockdown cells. Our analyses showed that CTHRC1 is regulated coordinately with fibronectin and integrin β3 by the pro-invasive and -angiogenic transcription factor NFATC2. We also found CTHRC1 to be a target of TFGβ and BRAF. These data highlight the importance of tumor stroma in melanoma progression. Furthermore, CTHRC1 was recognized as an important mediator of melanoma cell migration and invasion, providing together with its regulators—NFATC2, TGFβ, and BRAF—attractive therapeutic targets against metastatic melanomas.

## INTRODUCTION

Cutaneous melanoma represents one of the most aggressive malignancies, with a high tendency to metastasize. Melanoma may develop directly from transformed melanocytes/melanocyte precursor cells or through a stepwise progression first forming a benign nevus [1]. The nevus cells may acquire dysplastic features through genetic changes and further develop into a radial growth phase primary melanoma, which usually grows horizontally and rarely metastasizes. Through additional genetic and epigenetic changes, a vertical growth phase (VGP) primary melanoma may form, carrying an increased risk for metastasis. A vast number of mutations, including those of *TERT*, *BRAF*, *NRAS*, *PTEN*, and *CDKN2A*, have been linked to the malignant transformation of melanocytes in subgroups of primary melanomas [2–4], rendering therapeutic approaches challenging. A better strategy might, therefore, consist of identifying possible molecular changes common to the development and progression of different primary melanoma subtypes, which could be targeted together with the oncogenic driver mutations.

The Breslow's thickness [5] of the primary tumor is considered the best independent prognostic indicator in melanoma and the most important factor in assessing the risk of developing metastatic disease [6]. However, a significant portion (6.7%) of patients with thin melanomas (Breslow's thickness of 0.51–1.0 mm) present with disseminated disease [7], and 3% to 6% of patients with primary melanomas <1 mm thickness die of their disease within five years of diagnosis [6]. These cases show that the metastatic potential of primary melanomas may not be predicted from the morphological features alone and the use of routine histologic prognostic factors is insufficient for melanoma staging. In recent years, lymphatic mapping with invasive sentinel lymph node biopsy has become the standard staging method for melanoma, and the status of the sentinel lymph nodes represents a strong predictor of survival [8]. However, a portion of patients with metastatic disease remains undetected by this method, since 6% to 17% of sentinel lymph node—negative patients die within five years of diagnosis [8], indicating the presence of false-negative results or an alternative route of metastasis, i.e., hematogenous spreading. The shortcomings of these prognostic factors are indicative of the urgent need for new, better markers capable of identifying (preferably already in the primary tumors) the features determining the course of disease. This would spare patients from unnecessary surgery and morbidity related to sentinel lymph node biopsy. Unraveling the molecular events related to melanoma progression should also provide rational targets for novel therapeutic approaches. This is particularly important given that metastatic melanoma remains extremely difficult to cure. Recently, new molecularly targeted therapies against metastatic melanoma have been developed, but the responses have thus far remained transient and incomplete [2], whereby survival rates for patients with distant metastases have not improved much [6, 9].

Herein, our approach to reveal the molecular mechanisms underlying the development and progression of melanomas was to compare the gene expression profiles of benign nevi, and non-metastatic and metastatic primary melanomas. In particular, we aimed to identify possible changes common to all primary melanomas and, since the tumor microenvironment is known to promote tumor development and progression, also to identify the gene expression changes occurring in the tumor stroma. We found several genes associated with inflammation, angiogenesis, and extracellular matrix (ECM) modification to be commonly upregulated during the development and spreading of melanoma. Of particular interest, we found that the collagen triple helix repeat containing 1 (*CTHRC1*) gene was overexpressed in metastatic primary melanomas, and a high expression of CTHRC1 mRNA was associated with a short survival. We found that CTHRC1 was expressed by melanoma cells, activated stromal fibroblasts, and blood vessel endothelial cells. Our functional analyses suggested that CTHRC1 is required for the migration and invasion of melanoma cells *in vitro*, and that it may participate in regulating the switch between proliferation and invasion *in vivo*. Further, we found that the expression of CTHRC1 is induced coordinately with fibronectin (FN1) and integrin β3 (ITGB3) by the pro-invasive and -angiogenic transcription factor NFATC2 (the nuclear factor of activated T-cells, cytoplasmic, calcineurin-dependent 2). In addition to NFATC2, we found that CTHRC1 expression was increased by TGFβ and oncogenic BRAF signaling, making CTHRC1 an attractive new target for therapy.

## RESULTS

### Microarray analysis of benign nevi and primary melanomas

We first sought to identify those genes involved in melanoma development by comparing the gene expression profiles of fresh or frozen benign nevus (*n* = 11) and primary melanoma tissue samples (*n* = 21) also containing the adjacent stromal compartment. We were particularly interested in finding any common changes accompanying the development of heterogeneous primary melanomas. Significance Analysis of Microarrays (SAM) identified 1547 probe sets representing 1058 genes overexpressed ≥1.5-fold (Supplementary Table S1) and 1042 probe sets representing 731 genes underexpressed ≥1.5-fold (Supplementary Table S2) in primary melanomas compared to benign nevi. To determine which processes and pathways are activated in primary melanomas, we aimed to identify Gene Ontology (GO) classes and KEGG pathways overrepresented in the gene list and found, among others, inflammatory response (GO:0006954, *P* = 5.7 × 10^−7^, associated with, for instance, chemokine receptor *CXCR4*, *TGFB1*, *S100A8*, and *S100A9*), blood vessel development (GO:0001568, *P* = 3.5 × 10^−6^, associated with *THY1*, *COL4A2*, *CYR61*, and *FN1*), cell motility (GO:0048870, *P* = 6.6 × 10^−6^, associated with *CTHRC1, VCAN, THBS1, PLAU*, and *ITGB2*), and ECM-receptor interaction (KEGG:04512, *P* = 2.0 × 10^−6^, associated with collagens, *TNC*, *FN1*, *CD36*, laminins, and thrombospondins) as highly significant biological processes enriched among the overexpressed genes. To identify possible markers differentiating between benign nevi and melanoma, we filtered the gene list for at least a fourfold difference in the mean expression levels between groups (shown in bold in Supplementary Table S1). Here, genes encoding S100A proteins (*S100A7*, *S100A8*, and *S100A9*), *MIR21*, *CTHRC1*, *CXCR4*, *RGS1*, *PRAME*, and *SPP1* were identified as particularly interesting potential melanoma or melanoma-associated markers. Based on our microarray analyses of primary melanoma cells and melanoma cell lines as well as the publicly available microarray data of melanoma cell lines (*n* = 34; E-GEOD-7152), all of these potential markers except the S100A proteins may be expressed by melanoma cells (data not shown).

We then compared the gene expression profiles of non-metastatic and metastatic primary melanomas to determine which genes are involved in the metastatic process and the progression of melanomas, and to identify the potential predictive markers for metastasis. We followed patients in the non-metastatic group for 53 to 90 months (median follow-up, 84.5 months) with no signs of disease progression, while all patients in the metastatic group developed metastases within 0 to 9 months (median, 0 months) after the primary melanoma excision. SAM resulted in 1050 probe sets representing 787 genes overexpressed ≥1.5-fold (Supplementary Table S3) and 1517 probe sets representing 1133 genes underexpressed ≥1.5-fold (Supplementary Table S4) in the metastatic primary melanomas. A query of the SAM-ordered probes using the Gene Set Enrichment Analysis (GSEA) tool revealed that genes involved in the epithelial—mesenchymal transition (a gene set in the Hallmark signatures collection of the Molecular Signature Database) were enriched among those genes overexpressed in the metastatic primary melanomas (normalized enrichment score of 3.96 and false discovery rate q-value of <0.001). Further, genes associated with cell adhesion (GO:0007155, *P* = 6.9 × 10^−10^, e.g., *VCAN*, *SPP1*, *FN1*, *NEDD9*, and *ITGB3*), locomotion (GO:0040011, *P* = 2.5 × 10^−9^, *TIMP1*, *VCAN*, *TNS3*, and *CTHRC1*), and vasculature development (GO:0001944, *P* = 3.4 × 10^−8^, *ITGA5, COL4A1, THY1, FN1*, and *CYR61*) were upregulated in metastatic primary melanomas. Among the most interesting, highly and significantly over-expressed genes in metastatic primary melanomas were *TIMP1*, *VCAN*, *SPP1*, and *CTHRC1*, all of which are expressed both by stromal fibroblasts and melanoma cells (data not shown).

To determine if any genes were related to both the development and progression of melanomas, we analyzed the above gene lists for common genes (Table 1). In addition to the four genes mentioned above, we found that many of the top genes encode proteins that reside within or modify the extracellular space/proteinaceous ECM or function in cell adhesion, migration, invasion, or angiogenesis, such as *SERPINH1* [10], *LGALS1* [11], *SULF1* [12], *COL4A1* [13], *THY1* [14], *MME* [15], *LOXL2* [16], and *ADAM12* [17]. In addition, we found that the transcription factor HEY1 was commonly upregulated. We also searched the gene expression profiles of primary melanomas for potential markers of poor prognosis using the SAM survival analysis. We identified several interesting candidate genes, which were also specifically upregulated in melanoma cells compared to normal melanocytes. Of these genes, *FN1*, *P4HA1*, *RHOC*, *SCRG1*, *S100B*, and *SERPINA3* were most significantly associated with a short survival (Table 2). Noteworthy, several genes with a prognostic value, including *FN1* and *P4HA1*, are known to be induced by hypoxia [18, 19]. *FN1* is also one of the genes overexpressed both in primary melanomas compared to benign nevi (2.2-fold) and in metastatic compared to non-metastatic primary melanomas (2.0-fold) (Table 1). We further compared the Kaplan-Meier survival rates of patients with primary melanomas showing low and high FN1 mRNA expression levels and found the survival times to differ highly significantly between patient groups (Supplementary Figure S1A).

**Table 1:** The most significantly over-expressed genes^a^ shared in comparisons of primary melanomas vs benign nevi and metastatic vs non-metastatic primary melanomas by Significance Analysis of Microarrays (SAM) (ordered by SAM score of metastatic vs non-metastatic primary melanomas)

Gene	Gene description	Probe set ID	Mean ± Sthev		Fold_b_	SAM Score_c_	Fold_b_
			Non-metastatic primary melanomas	Metastatic primary melanomas	Metastatic vs non-metastatic	Metastatic vs non-metastatic	Melanomas vs nevi
*TIMP1*	TIMP metallopeptidase inhibitor 1	201666_at	1854 ± 833	5666 ± 2639	3.1	3.863	2.3
*VCAN*	Versican	204620_s_at	1047 ± 434	2466 ± 943	2.4	3.788	2.8
*SPP1*	Secreted phosphoprotein 1	209875_s_at	323 ± 218	4053 ± 3159	12.6	3.258	50.2
*CTHRC1*	Collagen triple helix repeat containing 1	225681_at	1307 ± 610	3127 ± 1450	2.4	3.240	4.1
*SERPINH1*	Serpin peptidase inhibitor, clade H, member 1	207714_s_at	536 ± 147	1114 ± 443	2.1	3.221	1.9
*LGALS1*	Lectin, galactoside-binding, soluble, 1	201105_at	3938 ± 1119	8473 ± 3897	2.2	3.144	1.6
*SULF1*	Sulfatase 1	212354_at	173 ± 58	483 ± 231	2.8	3.093	3.1
*COL4A1*	Collagen, type IV, alpha 1	211981_at	496 ± 222	953 ± 336	1.9	3.011	3.0
*THY1*	Thy-1 cell surface antigen	213869_x_at	347 ± 116	659 ± 242	1.9	2.869	2.6
*MME*	Membrane metallo-endopeptidase	203435_s_at	185 ± 50	401 ± 165	2.2	2.825	2.1
*LOC541471 / NCRNA00152*	Hypothetical LOC541471 / non-protein coding RNA 152	225799_at	448 ± 237	1140 ± 623	2.5	2.786	4.7
*C13orf18*	Chromosome 13 open reading frame 18	219471_at	184 ± 74	363 ± 124	2.0	2.763	1.7
*HEY1*	Hairy/enhancer-of-split related with YRPW motif 1	44783_s_at	564 ± 353	1720 ± 1093	3.1	2.762	2.7
*LOXL2*	Lysyl oxidase-like 2	202998_s_at	211 ± 88	416 ± 147	2.0	2.756	2.0
*IGKC*	Immunoglobulin kappa constant	221651_x_at	2934 ± 3593	8942 ± 5389	3.0	2.739	8.4
*A2M*	Alpha-2-macroglobulin	217757_at	2025 ± 800	3437 ± 1277	1.7	2.687	2.8
*ADAM12*	ADAM metallopeptidase domain 12	226777_at	63 ± 35	283 ± 183	4.5	2.672	6.1
*PARVB*	Parvin, beta	37966_at	250 ± 78	490 ± 198	2.0	2.660	1.5
*OLFML2B*	Olfactomedin-like 2B	213125_at	217 ± 124	457 ± 190	2.1	2.600	2.8
*SLC2A3*	Solute carrier family 2, member 3	202499_s_at	136 ± 69	446 ± 285	3.3	2.594	3.7
*IGHG1 / G2 / M / V4-31*	Immunoglobulin heavy constant gamma 1 / gamma 2 / mu / variable 4-31	211430_s_at	2376 ± 3258	8114 ± 5601	3.4	2.585	21.7
*ARPC1B*	Actin related protein 2/3 complex, subunit 1B	201954_at	1341 ± 451	3177 ± 1927	2.4	2.566	2.1
*DAB2*	Disabled homolog 2, mitogen-responsive phosphoprotein	201279_s_at	730 ± 401	1528 ± 764	2.1	2.559	1.9
*NES*	Nestin	218678_at	504 ± 251	1201 ± 691	2.4	2.552	1.6
*RNASE1*	Ribonuclease, RNase A family, 1 (pancreatic)	201785_at	1494 ± 829	3831 ± 2434	2.6	2.551	2.7
*C20orf112*	Chromosome 20 open reading frame 112	225224_at	213 ± 70	470 ± 233	2.2	2.532	1.7
*KDELR3*	KDEL (Lys-Asp-Glu-Leu) endoplasmic reticulum protein retention receptor 3	204017_at	372 ± 118	657 ± 256	1.8	2.520	2.0
*THBS1*	Thrombospondin 1	201108_s_at	154 ± 48	274 ± 81	1.8	2.491	2.1
*VSIG4*	V-set and immunoglobulin domain containing 4	204787_at	187 ± 97	553 ± 362	3.0	2.470	2.1
*SLC20A1*	Solute carrier family 20, member 1	201920_at	499 ± 239	1208 ± 739	2.4	2.461	3.8
*RRM2*	Ribonucleotide reductase M2	209773_s_at	303 ± 140	693 ± 389	2.3	2.434	3.8
*EGR1*	Early growth response 1	227404_s_at	2576 ± 936	5552 ± 3324	2.2	2.417	1.9
*TMEM158*	Transmembrane protein 158	213338_at	175 ± 42	630 ± 484	3.6	2.405	2.7
*FN1*	Fibronectin 1	210495_x_at	3032 ± 1186	6006 ± 3292	2.0	2.402	2.2
*CCL4*	Chemokine (C-C motif) ligand 4	204103_at	225 ± 111	557 ± 338	2.5	2.360	3.9
*IGKC / IGKV1-5*	Immunoglobulin kappa constant / variable 1-5	214836_x_at	600 ± 548	1766 ± 1267	2.9	2.360	4.6
*PLAUR*	Plasminogen activator, urokinase receptor	210845_s_at	192 ± 88	499 ± 315	2.6	2.336	2.4
*FCGR2A*	Fc fragment of IgG, low affinity IIa, receptor	203561_at	97 ± 54	490 ± 425	5.1	2.333	4.9
*CALU*	Calumenin	200755_s_at	702 ± 231	1251 ± 591	1.8	2.317	2.0
*CDKN3*	Cyclin-dependent kinase inhibitor 3	209714_s_at	157 ± 57	291 ± 109	1.9	2.313	3.0
*SH2B3*	SH2B adaptor protein 3	203320_at	287 ± 145	593 ± 307	2.1	2.306	2.3
*RAB20*	RAB20, member RAS oncogene family	219622_at	198 ± 64	562 ± 395	2.8	2.297	1.9
*ATP6V0E2*	ATPase, H+ transporting V0 subunit e2	213587_s_at	340 ± 92	751 ± 457	2.2	2.272	1.8
*IGLC7 / IGLV1-44*	Immunoglobulin lambda constant 7 / variable 1-44	215379_x_at	655 ± 752	2434 ± 2071	3.7	2.266	11.6
*PLA2G16*	Phospholipase A2, group XVI	209581_at	219 ± 145	408 ± 152	1.9	2.244	2.9
*GRB10*	Growth factor receptor-bound protein 10	209409_at	217 ± 76	463 ± 257	2.1	2.234	1.8
*IGLV1-44 / LOC100290481*	Immunoglobulin lambda variable 1-44 / immunoglobulin lambda light chain-like	214677_x_at	1483 ± 1970	5704 ± 5106	3.8	2.196	19.4
*CYR61*	Cysteine-rich, angiogenic inducer, 61	201289_at	350 ± 93	740 ± 451	2.1	2.179	3.3
*PCOLCE*	Procollagen C-endopeptidase enhancer	202465_at	726 ± 289	1332 ± 712	1.8	2.142	2.1
*CHN1*	Chimerin (chimaerin) 1	212624_s_at	247 ± 101	457 ± 216	1.8	2.130	2.8
*PAPSS2*	3′-phosphoadenosine 5′-phosphosulfate synthase 2	203058_s_at	159 ± 74	335 ± 180	2.1	2.113	2.6
*FCGR2C*	Fc fragment of IgG, low affinity IIc, receptor	210992_x_at	225 ± 90	582 ± 427	2.6	2.095	2.0
*MCAM*	Melanoma cell adhesion molecule	209086_x_at	326 ± 62	649 ± 387	2.0	2.079	1.6
*CCL2*	Chemokine (C-C motif) ligand 2	216598_s_at	385 ± 206	1346 ± 1242	3.5	2.076	4.6
*CCR1*	Chemokine (C-C motif) receptor 1	205099_s_at	109 ± 74	391 ± 333	3.6	2.064	4.0
*SLC2A14 / SLC2A3*	Solute carrier family 2, member 14 / solute carrier family 2, member 3	216236_s_at	272 ± 71	536 ± 311	2.0	2.050	1.8
*FKBP11*	FK506 binding protein 11	219118_at	269 ± 119	605 ± 404	2.2	2.047	3.0
*BCL2A1*	BCL2-related protein A1	205681_at	292 ± 306	682 ± 424	2.3	2.047	3.6
*IGLL3P*	Immunoglobulin lambda-like polypeptide 3, pseudogene	215946_x_at	175 ± 104	446 ± 317	2.5	2.038	2.8
*BST2*	Bone marrow stromal cell antigen 2	201641_at	297 ± 145	660 ± 440	2.2	2.037	2.2
*EMILIN2*	Elastin microfibril interfacer 2	224374_s_at	272 ± 136	593 ± 382	2.2	2.034	1.8

a Only annotated genes and the first probe set for each gene are shown. Full gene list is available by request.

b Genes with ≥2-fold change at least in one of the comparisons are shown.

c The FDR q-value for each was <0.001.

**Table 2:** Gene expression in primary melanomas most significantly associated with short survival by SAM survival analysis^a^

Gene	Gene description	Probe set ID	SAM_b_ Score	Fold
				Melanoma cells vs normal melanocytes_c_
*PLA1A*	Phospholipase A1 member A	219584_at	4.572	0.09
*CNP*	2′,3′-cyclic nucleotide 3′ phosphodiesterase	208912_s_at	4.369	0.78
*FN1*	Fibronectin 1	211719_x_at	4.265	4.26
*ATP1A1*	ATPase, Na+/K+ transporting, alpha 1 polypeptide	220948_s_at	4.247	0.77
*P4HA1*	Prolyl 4-hydroxylase, alpha polypeptide I	207543_s_at	4.147	4.29
*RHOC*	Ras homolog family member C	200885_at	4.131	2.45
*SCRG1*	Stimulator of chondrogenesis 1	205475_at	3.997	14.61
*SLC27A3*	Solute carrier family 27 (fatty acid transporter), member 3	222217_s_at	3.891	0.15
*S100B*	S100 calcium binding protein B	209686_at	3.871	4.62
*SERPINA3*	Serpin peptidase inhibitor, clade A (alpha-1 antiproteinase, antitrypsin), member 3	202376_at	3.858	37.16
*DUSP4*	Dual specificity phosphatase 4	226034_at	3.745	1.16
*CD63*	CD63 molecule	200663_at	3.738	0.68
*DUSP6*	Dual specificity phosphatase 6	208892_s_at	3.695	22.75
*ELOVL2*	ELOVL fatty acid elongase 2	213712_at	3.642	1.56
*SORT1*	Sortilin 1	212807_s_at	3.638	0.15
*SHC1*	SHC (Src homology 2 domain containing) transforming protein 1	214853_s_at	3.625	2.31
*BIN3*	Bridging integrator 3	222199_s_at	3.547	2.16
*MFI2*	Antigen p97 (melanoma associated) identified by monoclonal antibodies 133.2 and 96.5	235911_at	3.530	0.61
*PACSIN2*	Protein kinase C and casein kinase substrate in neurons 2	201651_s_at	3.528	1.18
*NES*	Nestin	218678_at	3.503	5.69
*PSRC1*	Proline/serine-rich coiled-coil 1	201896_s_at	3.497	2.22
*USP54*	Ubiquitin specific peptidase 54	227334_at	3.489	0.33
*ETV5*	Ets variant 5	203349_s_at	3.464	1.85
*CDK2AP1*	Cyclin-dependent kinase 2 associated protein 1	201938_at	3.462	1.31
*APOC2 / APOC4*	Apolipoprotein C-II / Apolipoprotein C-IV / APOC4-APOC2 readthrough (NMD candidate)	204561_x_at	3.422	1.00
*ARHGEF40*	Rho guanine nucleotide exchange factor (GEF) 40	220326_s_at	3.393	0.86
*CNN3*	Calponin 3, acidic	201445_at	3.358	2.46
*SPRY4*	Sprouty homolog 4 (Drosophila)	221489_s_at	3.344	12.73
*PLOD2*	Procollagen-lysine, 2-oxoglutarate 5-dioxygenase 2	202619_s_at	3.314	3.99
*SDC3*	Syndecan 3	202898_at	3.295	4.43
*GNG5*	Guanine nucleotide binding protein (G protein), gamma 5	207157_s_at	3.288	0.89
*HHATL*	Hedgehog acyltransferase-like	223572_at	3.282	1.06
*C8orf44-SGK3 / SGK3*	C8orf44-SGK3 readthrough / Serum/glucocorticoid regulated kinase family, member 3	220038_at	3.275	1.01
*CDH19*	Cadherin 19, type 2	206898_at	3.269	1.04
*BIRC7*	Baculoviral IAP repeat containing 7	220451_s_at	3.255	0.65
*VEGFA*	Vascular endothelial growth factor A	212171_x_at	3.242	2.85
*GAPDHP73*	Glyceraldehyde-3-phosphate dehydrogenase pseudogene 73	234954_at	3.231	4.36
*NPTX2*	Neuronal pentraxin II	213479_at	3.225	4.97
*ARPC1B*	Actin related protein 2/3 complex, subunit 1B, 41kDa	201954_at	3.221	1.56
*GANAB*	Glucosidase, alpha; neutral AB	211934_x_at	3.207	1.82
*SPRY2*	Sprouty homolog 2 (Drosophila)	204011_at	3.201	6.48
*ODC1*	Ornithine decarboxylase 1	200790_at	3.196	6.31
*SH2B3*	SH2B adaptor protein 3	203320_at	3.144	9.22
*DCBLD2*	Discoidin, CUB and LCCL domain containing 2	224911_s_at	3.133	2.36
*LEF1*	Lymphoid enhancer-binding factor 1	221558_s_at	3.131	2.05
*PSMB4*	Proteasome (prosome, macropain) subunit, beta type, 4	202243_s_at	3.125	0.95
*TIMP3*	TIMP metallopeptidase inhibitor 3	201147_s_at	3.121	27.56
*CSPG4*	Chondroitin sulfate proteoglycan 4	214297_at	3.107	5.71
*ACSL3*	Acyl-CoA synthetase long-chain family member 3	201662_s_at	3.104	4.65
*MESDC1*	Mesoderm development candidate 1	223264_at	3.102	0.69
*INPP5F*	Inositol polyphosphate-5-phosphatase F	203607_at	3.074	2.60
*ST3GAL6*	ST3 beta-galactoside alpha-2,3-sialyltransferase 6	210942_s_at	3.065	0.34
*COL9A3*	Collagen, type IX, alpha 3	204724_s_at	3.059	10.29
*APOD*	Apolipoprotein D	201525_at	3.025	0.07
*TBX2*	T-box 2	40560_at	3.003	1.02
*FKBP11*	FK506 binding protein 11, 19 kDa	219117_s_at	3.000	0.51
*HYOU1*	Hypoxia up-regulated 1	200825_s_at	2.998	3.21
*SRGAP1*	SLIT-ROBO Rho GTPase activating protein 1	227484_at	2.997	0.97
*TYR*	Tyrosinase	206630_at	2.988	0.02
*CHURC1-FNTB / FNTB*	CHURC1-FNTB readthrough / Farnesyltransferase, CAAX box, beta	225851_at	2.978	1.49
*PLEKHB1*	Pleckstrin homology domain containing, family B member 1	209504_s_at	2.976	0.93
*CHST11*	Carbohydrate (chondroitin 4) sulfotransferase 11	226372_at	2.971	2.52
*PLAT*	Plasminogen activator, tissue	201860_s_at	2.968	12.24
*SCCPDH*	Saccharopine dehydrogenase (putative)	201825_s_at	2.963	1.44
*POPDC3*	Popeye domain containing 3	219926_at	2.953	0.42
*SOX10*	SRY (sex determining region Y)-box 10	209842_at	2.948	0.68
*ZMAT3*	Zinc finger, matrin-type 3	219628_at	2.947	0.37
*PKM*	Pyruvate kinase, muscle	201251_at	2.931	4.13
*CDKN2C*	Cyclin-dependent kinase inhibitor 2C (p18, inhibits CDK4)	204159_at	2.928	1.45
*MMP16*	Matrix metallopeptidase 16 (membrane-inserted)	223614_at	2.918	13.33
*MCAM / MIR6756*	Melanoma cell adhesion molecule / MicroRNA 6756	210869_s_at	2.910	12.08
*SERPINE1*	Serpin peptidase inhibitor, clade E, member 1	202627_s_at	2.908	3.15
*PLOD3*	Procollagen-lysine, 2-oxoglutarate 5-dioxygenase 3	202185_at	2.905	1.74
*GJB1*	Gap junction protein, beta 1, 32kDa	204973_at	2.900	0.87
*KCNN4*	Potassium intermediate/small conductance calcium-activated channel, subfamily N, member 4	204401_at	2.893	5.47
*SHISA2*	Shisa family member 2	230493_at	2.889	2.87
*BACE2*	Beta-site APP-cleaving enzyme 2	217867_x_at	2.875	0.61
*S100A1*	S100 calcium binding protein A1	205334_at	2.860	0.68
*IGFBP2*	Insulin-like growth factor binding protein 2, 36kDa	202718_at	2.857	16.92
*UPP1*	Uridine phosphorylase 1	203234_at	2.857	1.40
*FLOT1*	Flotillin 1	208749_x_at	2.847	0.57
*C1orf85*	Chromosome 1 open reading frame 85	225401_at	2.846	0.23
*CNPY2*	Canopy FGF signaling regulator 2	209797_at	2.835	0.83

a Only annotated genes and the first probe set for each gene are shown. Full gene list is available by request.

b The FDR q-value for each was <0.001.

c Mean expression of WM239 and WM793 melanoma cells vs that of 42V melanocytes.

### CTHRC1 expression in different cell types

**Figure 1:**
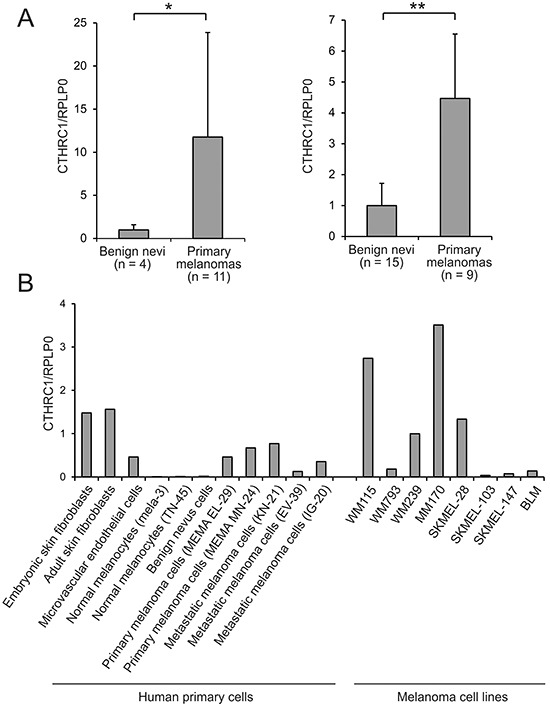
CTHRC1 mRNA expression in benign and malignant melanocytic lesions and cells, and other cell types **A.** Relative CTHRC1 expression levels in benign nevi and primary melanomas in two independent sample sets. CTHRC1 and RPLP0 cDNA levels were measured using qRT-PCR in triplicate for each sample. Bars represent standard deviations. *P = 0.0147, **P = 0.000907. **B.** CTHRC1 expression in cultures of different primary normal and melanoma cells, and melanoma cell lines. CTHRC1 and RPLP0 cDNA levels were measured using qRT-PCR in triplicate for each sample. CTHRC1/RPLP0 levels are shown relative to that of WM239.

*CTHRC1* is another interesting gene significantly overexpressed in primary melanomas compared to benign nevi (4.1-fold) and in metastatic compared to non-metastatic primary melanomas (2.4-fold) (Table 1). We confirmed the overexpression of CTHRC1 mRNA in primary melanomas compared to benign nevi using quantitative RT-PCR (qRT-PCR) in a subset of the microarray samples (Breslow's thickness: mean = 10.6 mm, median = 6.7 mm) as well as in an independent sample set (Breslow's thickness: mean = 4.1 mm, median = 4.0 mm), finding 11.8-fold and 4.7-fold differences, respectively, in the expression levels between groups (Figure 1A). Since we found that CTHRC1 was further overexpressed in metastatic primary melanomas, we sought to determine if CTHRC1 expression was associated with patient survival. Despite the limited sample size, we found a significant association between a shorter survival time and a high CTHRC1 mRNA expression in primary melanomas (Supplementary Figure S1B).

**Figure 2:**
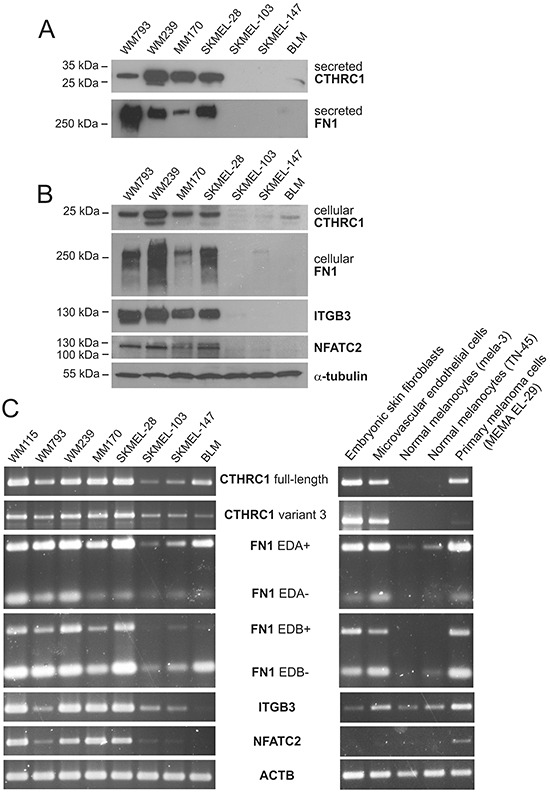
Expression of CTHRC1 and genes coordinately expressed with CTHRC1 in melanoma cell lines **A.** Western blot analysis of CTHRC1 and FN1 protein levels in conditioned media of a panel of melanoma cell lines. **B.** Western blot analysis of CTHRC1, FN1, ITGB3, and NFATC2 in cellular lysates of melanoma cell lines. Alpha-tubulin was used as the loading control. **C.** Expression of full-length and variant 3 CTHRC1, EDA+/− and EDB +/− FN1, ITGB3, and NFATC2 in a panel of melanoma cell lines and in different primary cells analyzed using semiquantitative RT-PCR. Actin (ACTB) was used as the control.

To determine which cell types are responsible for producing CTHRC1 in melanoma, we first used qRT-PCR to analyze the expression levels of CTHRC1 mRNA in primary human melanocytes, benign nevus cells, and melanoma cells isolated from primary tumors and lymph node metastases, as well as in primary human fibroblasts and endothelial cells. Among the primary cells, we found that both embryonic and adult skin fibroblasts showed the highest expression of CTHRC1 (Figure 1B). The cells from primary and metastatic melanomas showed a variable but higher (46-fold) CTHRC1 expression than normal melanocytes or benign nevus cells, in which CTHRC1 expression was virtually absent. The microvascular endothelial cells, in turn, expressed CTHRC1 mRNA at a moderate level similar to the melanoma cells. Our analyses of the established melanoma cell lines from VGP primary melanomas (WM115 and WM793) and melanoma metastases (WM239, MM170, SKMEL-28, SKMEL-103, SKMEL-147, and BLM) revealed that they expressed variable levels of CTHRC1 mRNA. The metastatic MM170 and SKMEL-28, as well as the VGP WM115 and metastatic WM239 melanoma cell lines (the latter two originating from the same patient) showed the highest CTHRC1 mRNA expression (Figure 1B). At the protein level, we found that WM239, MM170, and SKMEL-28 cells secreted the highest amounts of CTHRC1 (Figure 2A). Analyses of cellular CTHRC1 protein levels yielded similar results (Figure 2B).

### CTHRC1 is expressed coordinately with FN1, ITGB3, and NFATC2 in cells of melanocytic origin

To reveal in which processes CTHRC1 may function, we first sought out to identify genes coordinately expressed with CTHRC1 in the microarray data from our panel of normal melanocytes, benign nevus cells, primary melanoma cells, and two melanoma cell lines (WM793 and WM239), as well as independently from a panel of 34 melanoma cell lines obtained from a data bank (E-GEOD-7152). Shared genes positively correlating with CTHRC1 expression in both sample groups comprised *FN1, GPR126, NFATC2*, and *ITGB3* (Supplementary Table S5). Using semiquantitative RT-PCR, we then analyzed the mRNA levels of FN1 (with primers amplifying FN1 with and without the extradomains A and B: EDA+, EDA-, EDB+, and EDB- FN1), ITGB3, and NFATC2 in WM115, WM793, WM239, MM170, SKMEL-28, SKMEL-103, SKMEL-147, and BLM melanoma cell lines. We found that these genes were expressed in a manner similar to CTHRC1 (Figure 2C). Interestingly, in cultures of primary embryonic fibroblasts, endothelial cells, melanocytes, and melanoma cells from a primary tumor, CTHRC1 and FN1 were expressed similarly at a high level in fibroblasts, endothelial cells, and melanoma cells, whereas the ITGB3 expression was high in endothelial and melanoma cells but very low in fibroblasts, while NFATC2 seemed to be expressed only in the melanoma cells among the cell types tested (Figure 2C). At the protein level, FN1, ITGB3, and NFATC2 were expressed in a manner similar to CTHRC1 in our panel of melanoma cell lines (Figure 2A, 2B).

We also studied which genes are coordinately upregulated with CTHRC1 in primary melanoma tissues. Using hierarchical clustering, we found that many genes encoding ECM proteins, such as *FN1*, *COL4A1*, *COL4A2*, *NID1*, and *EMILIN2*, clustered with *CTHRC1* (Supplementary Figure S2). Using hypergeometric testing for GO classes and KEGG pathways, we found that those genes clustering with *CTHRC1* were associated with various biological processes and pathways, of which blood vessel development (GO:0001568, *P* = 9.1 × 10^−6^) and locomotion (GO:0040011, *P* = 9.1 × 10^−6^) proved most significant, along with ECM-receptor interaction (KEGG:04512, *P* = 4.0 × 10^−6^) and focal adhesion (KEGG:04510, *P* = 0.00012).

### CTHRC1 is overexpressed in melanoma tissue by both tumor and stromal cells

**Figure 3:**
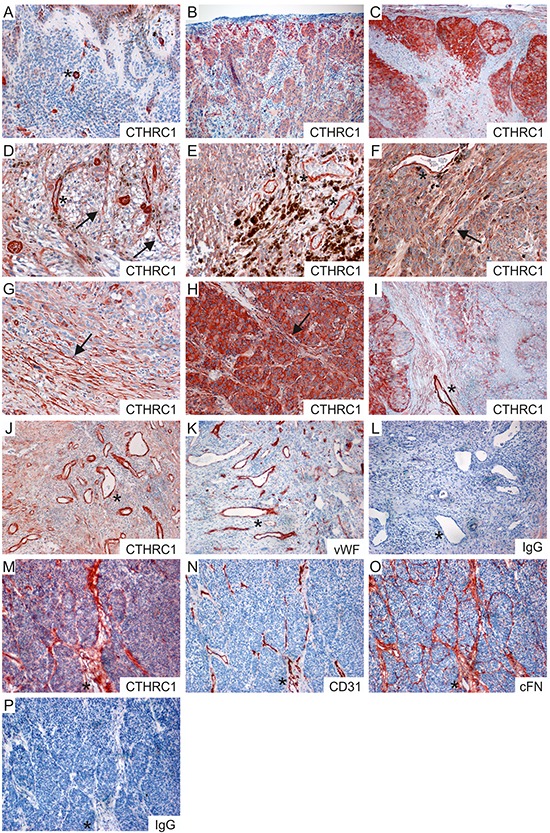
Immunohistochemical staining of CTHRC1, vWF, CD31, and cFN in primary melanomas and melanoma metastases **A–C.** Representative stainings of CTHRC1 in a benign nevus (A), a primary melanoma (B), and a melanoma lymph node macrometastasis (C). **D–I.** Examples of primary melanomas (D–F) and melanoma lymph node metastases (G–I) showing different levels of CTHRC1 staining in the melanoma cells. Note that strong staining was also seen in the irregular tumor blood vessels (D, E, F, I; marked with asterisks) and in the fibroblasts (marked with arrows) surrounding the melanoma cell nests (D, F, H) and at the invasion fronts (G). **J–L.** Sections from a primary melanoma stained with antibodies recognizing CTHRC1 (J), vWF (K), and with a normal rabbit IgG control antibody (L). **M–P.** Consecutive frozen sections from a primary melanoma stained with antibodies recognizing CTHRC1 (M), CD31 (N), and cFN (O), and with a normal rabbit IgG control antibody (P). (A-P) Positive immunostaining is shown in red (AEC). Original magnification 100x (B, C, I–P) and 200x (A, D–H). Examples of fibroblasts are marked with arrows, while asterisks indicate blood vessels.

Next, using immunohistochemistry we analyzed the protein expression levels and tissue distribution of CTHRC1 in benign nevi (*n* = 15), primary melanomas (*n* = 16), and melanoma lymph node metastases (*n* = 19). In benign nevi (Figure 3A), CTHRC1 showed faint to modest staining, while in primary melanomas and melanoma metastases the CTHRC1 staining varied from moderate to strong (Figure 3B, 3C). We found that the intensity of CTHRC1 staining in melanoma cells varied in individual melanoma tissue samples, with a stronger staining usually seen at the periphery of the tumor (i.e. in invasion fronts) and in areas where the melanoma cells contacted stromal cells (Figure 3D–3I). We also found strong CTHRC1 staining in fibroblasts surrounding the melanoma cell nests, and in the intimal layer of forming and newly-formed tumor blood vessels, as well as in the walls of small, normal blood vessels (Figure 3A–3I and Supplementary Figure S3), visualized using staining with the endothelial cell marker von Willebrand factor (vWF; Figure 3J, 3K). Furthermore, we also saw strong staining in the adventitia of large arteries (not shown). These data support our pathway analysis results, suggesting that *CTHRC1* and genes correlating with its expression are involved in the development of blood vessels.

To confirm the CTHRC1 staining pattern, we additionally stained frozen sections from three primary melanomas with another antibody that recognized the native form of the CTHRC1 protein in Western blotting. Again, we found that CTHRC1 localized in both the melanoma cells and stromal fibroblasts (Figure 3M). In addition, CTHRC1 localized with the endothelial cell marker CD31 in blood vessels (Figure 3M, 3N). Since FN1 mRNA expression correlated with that of CTHRC1 in both melanoma cell cultures and primary melanoma tissues, we stained consecutive sections with a monoclonal antibody detecting cellular fibronectin (cFN; EDA+ FN1), finding that CTHRC1 and cFN co-localized in the fibroblasts or fibrillar structures surrounding melanoma cell nests and in blood vessels (Figure 3M–3O).

### Characterization of the CTHRC1 protein

Since the function of CTHRC1 remains mostly unknown, we first explored the structural and molecular properties of the CTHRC1 protein. When we immunoblotted CTHRC1 under nonreducing conditions, we found that the secreted CTHRC1 existed primarily as a dimer (~56 kDa) and a trimer (~84 kDa) as well as multimers of the trimeric CTHRC1 (~168 kDa and ~252 kDa) (Supplementary Figure S4A). The cellular CTHRC1 existed mostly as a dimer and a trimer. When we analyzed CTHRC1 under reducing conditions, the molecular weight of the secreted CTHRC1 appeared to be larger than that of the cellular CTHRC1, the weights being approximately 30 kDa and 26 kDa, respectively (Supplementary Figure S4A). We then aimed to resolve whether the larger molecular weight is a result of some post-translational modification or whether the secreted and cellular CTHRC1 represent different isoforms. According to the Ensembl Database, four different isoforms of CTHRC1 exist, with the molecular weights of approximately 26.2 kDa (full-length, NP_612464.1), 12.3 kDa (variant 2), 24.8 kDa (variant 3, NP_001243028.1), and 16 kDa (variant 4). Compared to the full-length protein, variant 3 contains an alternate 5′ exon and a translation start site, and no signal sequence for extracellular secretion. Since the qRT-PCR expression assay we used measures both the full-length and variant 3 mRNA levels, we investigated the expression of these two splice variants separately using semiquantitative RT-PCR. We found that both the full-length and variant 3 mRNAs were expressed in melanoma cell lines as well as in embryonic fibroblasts and microvascular endothelial cells (Figure 2C), although the variant 3 mRNA was expressed at a markedly lower level. We then confirmed the PCR products through sequencing. To determine if variant 3 accounts for the cellular CTHRC1 protein in melanoma cells, we knocked down the CTHRC1 expression with small interfering RNAs (siRNAs) specific to the full-length or variant 3 mRNA and with siRNAs targeting both splice variants. SiRNAs targeting the variant 3 CTHRC1 significantly reduced the variant 3 mRNA (Supplementary Figure S4B), but we found no effect in the cellular protein levels of CTHRC1 (Supplementary Figure S4C). However, both the cellular and secreted CTHRC1 protein levels were reduced by siRNAs targeting the full-length CTHRC1 (Supplementary Figure S4C), suggesting that, despite the difference in molecular weight, the full-length CTHRC1 accounts for both the secreted and cellular CTHRC1, while the variant 3 protein remains relatively unexpressed.

Because the CTHRC1 protein contains a glycosylation site, we then set out to determine whether glycosylation is responsible for the different migration rates of secreted and cellular CTHRC1 in SDS-PAGE. Treating the secreted proteins and cellular lysates with *N*-glycosidase F resulted in similar migration rates for both pools of CTHRC1 (Supplementary Figure S4D), suggesting that CTHRC1 is further glycosylated prior to secretion.

### CTHRC1 is required for the migration and invasion of melanoma cells

**Figure 4:**
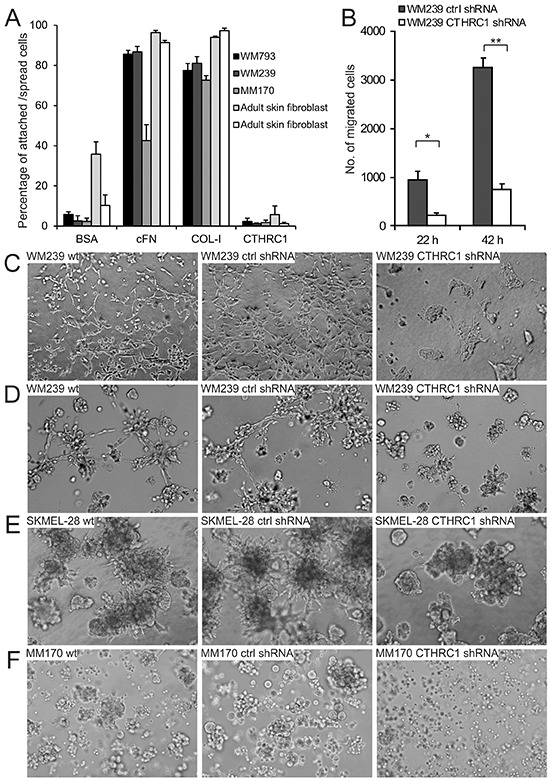
Effect of CTHRC1 on cell adhesion, migration, and invasion **A.** Adhesion of WM793, WM239, and MM170 melanoma cell lines and primary human fibroblasts on surfaces coated with cFN, COL-I, and CTHRC1. BSA was used as the control. Values consists of means ± standard deviations of three replicates. **B.** The effect of CTHRC1 on cell migration. The number of WM239 control (ctrl) and CTHRC1-knockdown cells migrated through transwell inserts after incubation for 22 and 42 hours. Values consist of means ± standard deviations of three replicates. *P = 0.002, **P < 0.0001. **C–F.** The effect of CTHRC1 on the invasive growth of melanoma cells in 3D gels. Culture of WM239 wild-type (wt), control, and CTHRC1-knockdown cells embedded between two layers of 3D collagen-I gel for four days (C) and between two layers of Matrigel for five days (D). Culture of SKMEL-28 (E) and MM170 (F) wt, control, and CTHRC1-knockdown cells embedded between two layers of Matrigel for eight days.

Since CTHRC1 overexpression has been found to enhance cell adhesion and migration [20, 21], we tested the adhesion of melanoma cells (WM793, WM239, and MM170) and adult human fibroblasts on surfaces coated with the recombinant full-length CTHRC1 protein produced in human cells. Unexpectedly, we found that CTHRC1 did not support the adhesion of cells (Figure 4A). We then tested the effect of endogenous CTHRC1 on cell adhesion by knocking down CTHRC1 expression in WM239 melanoma cells by short hairpin RNAs (shRNAs) targeting all CTHRC1 variants (for the level of knockdown, see Supplementary Figure S5A–5C). We found no differences in the cell adhesion properties of the control and CTHRC1-knockdown cells plated on uncoated plastic surfaces or surfaces coated with ECM proteins such as cFN, collagen I (COL-I), and laminin (data not shown). However, when we tested the effect of endogenous CTHRC1 on cell migration through uncoated transwell inserts, the CTHRC1-knockdown cells showed significantly decreased motility (4.4-fold after 22 hours; 4.3-fold after 42 hours) compared to the control cells (Figure 4B).

To further test the effect of CTHRC1 on melanoma cell migration and invasion in a three-dimensional (3D) environment more closely mimicking the *in vivo* situation, we plated the cells between two layers of COL-I gel or Matrigel. In these assays, the migration and invasion capability of the WM239 CTHRC1-knockdown cells was markedly impaired compared to the wild-type or control shRNA cells (Figure 4C, 4D). It is also notable that knockdown of CTHRC1 resulted in a change in cell morphology, i.e. from the spindle shape (typical of migrating cells) of the parental cells to an epithelioid morphology (Figure 4C). It should also be noted here, that the growth rate of the WM239 CTHRC1-knockdown cells in 2D was not altered or was slightly increased compared to the wild-type or the control shRNA cells (data not shown). Further, knocking down CTHRC1 expression also inhibited the invasive growth of SKMEL-28 and MM170 melanoma cells embedded in thick 3D Matrigel (Figure 4E, 4F; for the levels of knockdown see Supplementary Figure S5).

### Regulation of CTHRC1 expression

**Figure 5:**
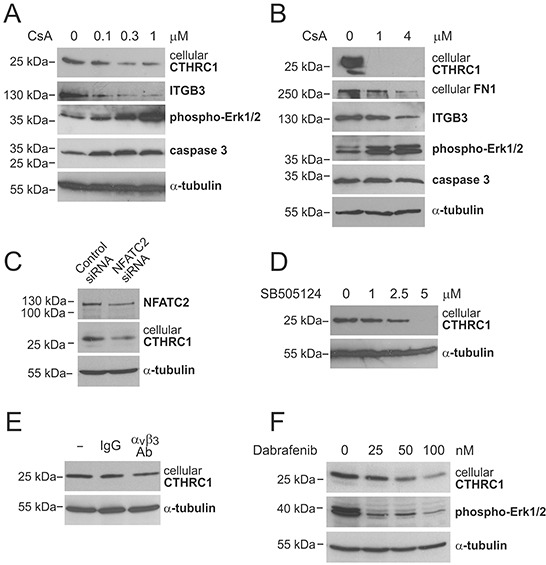
Regulation of CTHRC1 expression **A** and **B.** Western blot analysis of CTHRC1 and other proteins of interest in WM239 cells treated without or with increasing concentrations of cyclosporin A (CsA) for 24 hours (A) and 48 hours (B). **C.** Western blot analysis of NFATC2 and CTHRC1 in WM239 control and NFATC2-knockdown cells 72 hours after siRNA transfection. **D.** Western blot analysis of CTHRC1 in WM239 cells treated without or with increasing concentrations of TGFβ-receptor I/ALK-5 inhibitor SB505124 for 48 hours. **E.** Western blot analysis of CTHRC1 in WM239 cells treated without or with 10 μg/ml control antibodies (IgG) and neutralizing antibodies to integrin α_v_β_3_ for 48 hours. **F.** Western blot analysis of CTHRC1 and phospho-Erk1/2 in WM239 cells treated without or with increasing concentrations of BRAF inhibitor Dabrafenib for 48 hours. Alpha-tubulin was used as the loading control.

Since we found that CTHRC1 is coordinately expressed with the transcription factor NFATC2 in melanoma cell lines, and the NFATC2 protein has been found to be expressed by melanoma cells *in vitro* and *in vivo* [22], we studied whether NFATC2 induces the expression of CTHRC1. We first treated WM239 melanoma cells with cyclosporin A (CsA), which inhibits calcineurin that, in turn, dephosphorylates and activates NFATs. Treatment with increasing concentrations of CsA resulted in a dose-dependent reduction in the expression of the CTHRC1 protein after 24 hours (Figure 5A). In addition, the protein levels of ITGB3 (the expression of which we found to correlate with that of CTHRC1) markedly decreased after treatment with CsA for 24 hours (Figure 5A). Likewise, the protein expression levels of FN1 (which were coordinately expressed with CTHRC1) were downregulated, although only after 48 hours of incubation with CsA (≥1 μM) (Figure 5B), possibly due to the longer half-life of this protein. We further found that low CsA concentrations (≤1 μM) increased the levels of phospho-Erk1/2 and uncleaved caspase-3 (32-kDa zymogen) (Figure 5A, 5B), indicating that the downregulation of CTHRC1 is not consequent to decreased cell viability. CsA treatment was also found to decrease CTHRC1 protein levels in MM170 melanoma cells (Supplementary Figure S6A). Because CsA inhibits the activity of all NFAT proteins, we additionally studied the effect of NFATC2 inhibition using specific siRNAs. The knockdown of the NFATC2 expression using these siRNAs remained incomplete, yet still resulted in a clear reduction (50%) of CTHRC1 protein expression (Figure 5C).

Because previous studies have reported that TGFβ increases CTHRC1 expression and that CTHRC1, in turn, inhibits TGFβ signaling in different cell types [20, 23], we examined the role of TGFβ in the regulation of CTHRC1 in melanoma cells by inhibiting TGFβ signaling with increasing concentrations of a specific TGFβ-receptor I/ALK-5 inhibitor SB505124. We found a dose-dependent reduction of the CTHRC1 protein levels in both WM239 and MM170 cells after 24 and 48 hours of inhibition (Figure 5D, Supplementary Figure S6B, and data not shown).

In addition, since we found that the expression of ITGB3 correlated with that of CTHRC1 in melanoma cells (Supplementary Table S5) and primary melanoma tissues (data not shown) (and we found no changes in the ITGB3 expression levels in CTHRC1-knockdown cells, data not shown), we examined whether CTHRC1 could be a downstream target of ITGB3 signaling. Indeed, incubating WM239 cells with neutralizing antibodies to α_v_β_3_ integrin for 48 hours resulted in a reduction in the CTHRC1 protein levels (Figure 5E).

Furthermore, since oncogenic BRAF has been found to activate NFAT signaling in melanoma [24], and we found that CTHRC1 is expressed at high levels mainly in BRAF mutant melanoma cell lines, we examined the effect of BRAF inhibition on CTHRC1 expression. Treating the WM239 cells (BRAF V600D mutant) with increasing concentrations of the BRAF inhibitor Dabrafenib for 48 hours resulted in a dose-dependent reduction of BRAF activity (measured by the reduced phospho-Erk1/2 levels) and the expression of the CTHRC1 protein (Figure 5F). Similarly, CTHRC1 protein levels were also decreased in MM170 cells (BRAF V600E mutant) after treatment with Dabrafenib for 24 hours (Supplementary Figure S6C).

### Gene expression changes in CTHRC1-knockdown cells

To unravel the molecular mechanisms of the CTHRC1 action, we analyzed the gene expression profiles of the WM239 control shRNA and CTHRC1-knockdown cells using microarray analyses. We found 41 probe sets representing 36 annotated genes downregulated (with ≥1.5-fold change and ≥100 difference in the expression values) in the CTHRC1-knockdown cells compared to the control shRNA cells (Supplementary Table S6). The most downregulated gene in the knockdown cells was *CTHRC1* itself with a 2.8-fold reduction in expression. The second most downregulated gene was aldo-keto reductase family 1, member C3 (*AKR1C3*), which has been reported to be overexpressed in several cancers [25–27], and to increase the migration and invasion of cervical cancer cells and to change the organization of their cytoskeleton [28]. Other downregulated genes included fatty acid binding protein 7 (*FABP7*), previously linked to the proliferation, migration, and invasion of tumor cells [29, 30], and cofilin 1 (CFL1), a central regulator protein of actin dynamics in migrating cells (reviewed in [31]). Furthermore, WNT5A, an activator of the non-canonical Wnt signaling pathway, also emerged among the downregulated genes.

We further confirmed the downregulation of CTHRC1, AKR1C3, WNT5A, and FABP7 mRNA expression levels in the CTHRC1-knockdown cells by qRT-PCR (Supplementary Table S7). To confirm the reproducibility of these results, we studied the expression levels of these genes in a separate knockdown experiment in WM239 cells. These CTHRC1-knockdown cells showed an even larger decrease in the mRNA levels of CTHRC1 and the other downregulated genes compared to those of the control shRNA cells (Supplementary Table S7) To further evaluate the generality of these findings, we analyzed the expression levels of these potential downstream targets of CTHRC1 in another melanoma cell line, MM170, with similar results (Supplementary Table S7). In particular, AKR1C3 was highly downregulated in the CTHRC1-knockdown cells.

### Effect of CTHRC1 knockdown on tumorigenesis of WM239 melanoma cells in nude mice

**Figure 6:**
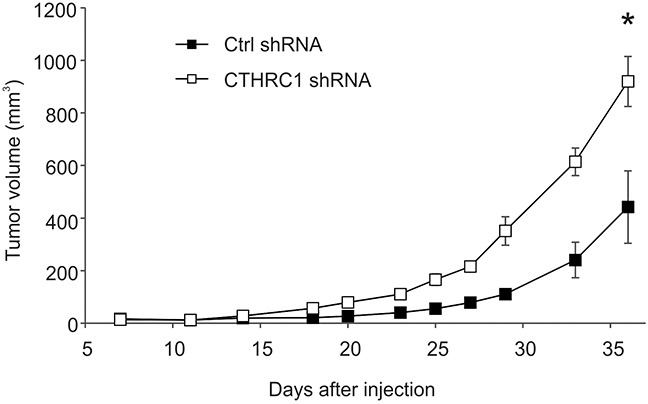
Growth curves of tumors induced by control (ctrl) shRNA-expressing and CTHRC1-knockdown WM239 cells in nude mice Cells (6 × 10^6^) were injected subcutaneously into the lower flank of the individual mice (six mice per group). Tumor volume was measured with a caliper two to three times per week. Bars represent standard deviations. *P < 0.00001.

To study the effect of CTHRC1 on tumorigenesis in nude mice, we injected WM239 CTHRC1-knockdown and control shRNA-expressing cells (6 × 10^6^) with or without Matrigel subcutaneously into the lower flanks of the mice. As CTHRC1 expression was clearly associated with tumor progression in clinical human melanomas, we unexpectedly found that the growth rate of CTHRC1-knockdown cells was increased compared to the control shRNA cells in two repeated experiments (Figure 6 and Supplementary Figure S7). When excised, the knockdown tumors were found to be markedly softer than the control tumors. First, we confirmed the knockdown of CTHRC1 mRNA expression levels by qRT-PCR analyses, which revealed about 10-fold decrease in expression (data not shown). Histologically, the tumors in both groups were heterogeneous (Supplementary Figure S8), similar to the parental WM239 cell line. The knockdown tumors showed consistently more necrotic areas, which, as a result of fluid accumulation, may contribute to the looser structure of the tissue and larger size of the tumors. We then studied other possible mechanisms for the increased size of the knockdown tumors by analyzing the proliferation and survival statuses of the cells. Immunostaining of the DNA synthesis marker, minichromosome maintenance complex component 7 (MCM7), appeared to show increased intensity in the CTHRC1-knockdown tumors as compared to the control tumors (Supplementary Figure S8E-S8H), regardless of the tumor size. Staining of the apoptosis marker, cleaved/active caspase 3, counterintuitively suggested also increased apoptosis in the CTHRC1-knockdown tumors but only locally beside the necrotic areas (Supplementary Figure S8I-8L). Whether this is due to reduced angiogenesis or other factors in the CTHRC1-knockdown cells remains to be seen.

We then stained the tumors for the melanoma marker S100, the expression/secretion levels of which are known to be associated with metastasis and poor patient prognosis [32, 33]. Interestingly, the CTHRC1-knockdown tumors showed a markedly decreased staining of S100 (Supplementary Figure S8M-S8P). Of additional interest, the smallest control tumor with a high CTHRC1 expression showed high invasive capability (muscle invasion) and formed a rapidly growing secondary tumor (data not shown).

## DISCUSSION

Melanoma, a highly aggressive and heterogeneous disease, continues to present a significant therapeutic challenge. In our research, we aimed to identify genes commonly associated with the development and progression of primary melanomas by screening the gene expression profiles of fresh/frozen benign nevi and non-metastatic and metastatic primary melanomas (difficult to obtain without interfering with diagnostics). The tissue samples also contained the associated stromal compartment, allowing us to identify changes occurring in the tumor microenvironment as well. Interestingly, we found several genes involved in the ECM modification, such as FN1 and P4HA1, which may be expressed by both melanoma and stromal cells, to be associated with metastasis and patient prognosis. Determining if these changes are associated with the formation of tubular FN1-, COL-I-, and tenascin C-containing fibrillar structures, which we have previously suggested to be invasion channels for melanoma cells [34, 35], remains of interest. In support of this idea, collagen prolyl hydroxylases have been found to promote the invasion and metastasis of breast cancer cells through collagen deposition [36]. Another important tumor-promoting process in the microenvironment is inflammation. Consistent with this, we found that the pro-inflammatory cytokines S100A8 and S100A9 (reviewed in [37]), as well as the chemokine receptor CXCR4, were highly and significantly upregulated in primary melanomas compared to benign nevi. The importance of S100A proteins in melanoma development has been less studied, but the expression of CXCR4 has been reported to predict poor prognosis in melanoma [38]. In addition to tumor cells, CXCR4 is expressed by several other cell types, including macrophages and endothelial cells, and the CXCL12—CXCR4 pathway is associated with tumor progression, metastasis, and angiogenesis (reviewed in [39]), also providing an attractive target for melanoma therapy. Another potential therapy target is MIR21, which we found to be highly upregulated in primary melanomas (significant upregulation validated using qRT-PCR, data not shown). The expression of MIR21 has further been shown to increase during melanoma progression and to predict poor survival [40]. Consistent with this, we found that both CXCR4 and MIR21 were upregulated in metastatic compared to non-metastatic primary melanomas. Interestingly, several protease inhibitors, such as TIMP1, TIMP3, and SERPINE2, were also upregulated in metastatic primary melanomas. Although protease inhibitors have been expected to display anti-tumorigenic properties, they have also been shown to play pro-tumorigenic roles and to be associated with poor prognosis in various cancers [41–45], warranting more detailed studies of their functions in melanoma as well.

Most interestingly, we found that many of the genes we have previously identified as upregulated in melanoma lymph node metastases [35, 46] were already upregulated in metastatic primary tumors, including *SPP1* and *CTHRC1* as well as the ECM protein-encoding genes *VCAN*, *LGALS1*, and *FN1*. Among these, we further found that SPP1, CTHRC1, LGALS1, and, most significantly, FN1 mRNA correlated with a poor survival (Table 2, Supplementary Figure S1, and data not shown), although these results must be validated in a larger data set. Interestingly, we found that CTHRC1 and FN1 mRNA expression correlated in primary melanocytes, nevus cells, and melanoma cells, as well as in melanoma cell lines and primary melanoma tissues. Other genes correlating with CTHRC1 expression in melanoma cell lines included the transcription factor NFATC2 and ITGB3. The coordinate expression of these genes may at least partly result from regulation by TGFβ, since all of them have been suggested to be TGFβ target genes [20, 47–49]. We have also found that TGFβ signaling is activated during the progression of melanomas [35]. It appears that NFATC2 may further increase the expression of CTHRC1, FN1, and ITGB3, given that we found their protein levels to decrease after treatment with CsA or specific NFATC2 siRNAs. Indeed, ITGB3 expression has recently been suggested to be increased by the calcineurin-NFAT signaling [50]. Furthermore, we found that the inhibition of αvβ3 integrin activity using neutralizing antibodies reduced CTHRC1 expression, suggesting a regulatory role for ITGB3 in CTHRC1 expression. ITGB3 has been suggested to play an important role in melanoma progression since it is overexpressed in VGP primary melanomas and in melanoma metastases [51], and its overexpression induces the conversion of melanoma cells from the radial to vertical growth phase [52]. Integrin αvβ3 functions as a receptor for many ECM proteins containing the classical integrin-recognition RGD motif, including vitronectin, FN1, fibrinogen, vWF, thrombospondin, and SPP1 [53–55]. Thus, elevated levels of FN1 may induce the coordinate expression of CTHRC1 in melanoma cells by activating the αvβ3 integrin receptor. The αvβ3 integrin receptor may also be activated by other ligands expressed by melanoma cells, such as SPP1, as we recently reported [56]. Interestingly, SPP1, one of the most highly upregulated genes in melanoma progression, has also been shown to promote osteoclast survival by activating calcineurin-NFAT signaling through integrin binding via its RGD motif [57].

CTHRC1 is overexpressed in many cancers, including gastric [58], pancreatic [21], hepatocellular [59], breast [60], and non-small cell lung cancer [61], as well as in melanoma [62]. In melanoma, CTHRC1 has previously been reported to be expressed by melanoma cells. However, we found that CTHRC1 is expressed in *in vitro* cultured cells and in human melanoma tissue samples by both melanoma and stromal cells. The stromal expression of CTHRC1 has also been noted in breast cancer, where CTHRC1 is expressed in breast cancer cells [60, 63] as well as in the stromal myoepithelial cells and myofibroblasts [64]. Originally, CTHRC1 was identified as a protein transiently expressed in injured arteries by fibroblasts of the remodeling adventitia and by smooth muscle cells of the neointima, and it was found to promote the migration of these cells *in vitro* [20]. Consistent with this, we found that the genes correlating with CTHRC1 expression in primary melanoma tissues were most significantly associated with blood vessel development and locomotion. We detected a high CTHRC1 expression in the tumor-associated fibroblasts/activated fibroblasts and in the intimal layer of aberrantly shaped immature tumor blood vessels. We also found staining in the walls and adventitia of normal blood vessels. Importantly, no obvious fibroblastic staining was found in the benign nevi, consistent with an earlier study reporting that fibroblast-like cells in normal tissues do not express CTHRC1 [65]. It thus appears that the upregulation of CTHRC1 mRNA in the primary melanomas compared to benign nevi primarily results from an increase in blood vessel formation and from the recruitment and activation of stromal fibroblasts. We should also note that the activation of fibroblasts is likely independent of the mutational status of melanomas cells [66]. As a result of this and the increased angiogenesis, CTHRC1 is likely to be universally increased in melanomas. In melanoma cells, the most intensive CTHRC1 staining was found in areas contacting stromal cells, suggesting that the interplay between melanoma cells and fibroblasts is important in inducing CTHRC1 expression. Furthermore, because CTHRC1 is a secreted protein, it may function as a paracrine factor in the tumor microenvironment, promoting the migration and invasion of many cell types regardless of the cell-type that has produced it.

Interestingly, the genes coordinately expressed with *CTHRC1*, i.e. *ITGB3*, *NFAT*, and *FN1*, have all been implicated in angiogenesis, suggesting that these molecules may be associated with the increased angiogenesis and blood vessel density detected in melanomas. In support of this idea, integrin αvβ3 has long been recognized as an important receptor in endothelial cells. ITGB3 forms a complex with VEGFR2 in a VEGF-dependent manner, leading to the phosphorylation of both receptors and the promotion of the adhesion, spreading, and migration of endothelial cells [67, 68]. VEGF has also been found to activate NFAT signaling in endothelial cells, and to promote endothelial cell migration and angiogenesis through NFAT signaling [69]. FN1, in turn, is essential in vascular morphogenesis [70], and both the EDA- and EDB-containing FN1 isoforms have been proposed as vascular markers in tumors [71, 72]. Furthermore, we have previously demonstrated that the EDA-containing FN1 (cFN) is upregulated in forming or newly formed blood vessels in melanoma lymph node metastases [35]. Here, we found that cFN co-localized with CTHRC1 in the blood vessels of primary melanoma tumors. It may also be noteworthy that we found neuropilin 2 (NRP2), a cell surface receptor for VEGF (reviewed in [73]) and TGFβ [74], to be coordinately expressed with CTHRC1 in melanoma cell lines (data not shown). This pro-angiogenic factor is expressed by tumor cells as well as vascular and lymphatic endothelial cells, and functions as an important mediator of melanoma and endothelial cell communication [75].

The function of CTHRC1 in melanoma remains largely unknown. The knockdown of CTHRC1 expression in melanoma cells has been found to decrease melanoma cell migration *in vitro* [62], a finding consistent with our results. Importantly, we further found that the knockdown of CTHRC1 inhibited the invasive growth of melanoma cells in 3D Matrigel and COL-I gels. How then does CTHRC1 exert its effect on cell migration and invasion? One of the genes that we found to be downregulated in the CTHRC1-knockdown cells was *WNT5A*. CTHRC1 has been suggested to bind WNT5A and stabilize the binding of Wnt to its receptor, thus activating Wnt signaling [76]. Consequently, the activation of Wnt signaling may create a positive feedback loop, where the expression of both WNT5A and CTHRC1 is maintained through increased signaling of the downstream protein kinase C [77] and calcineurin—NFAT pathways [78, 79], respectively. In addition, the *CFL1* gene downregulated in the CTHRC1-knockdown cells may be activated by a calcineurin-dependent mechanism [80]. Both genes are thus potential effectors of CTHRC1-induced cell migration and invasion, and their importance and functions in these processes remain interesting subjects for future research.

In our *in vivo* xenograft experiments in nude mice, we found that knockdown of CTHRC1 in WM239 melanoma cells increased tumor growth. It is recently recognized that cancer cells cannot proliferate and invade at the same time [81, 82]. Thus, one explanation to our rather unexpected result in nude mice may be that CTHRC1 may regulate the switch from proliferation to invasion, as we found CTHRC1 to promote invasion but not proliferation *in vitro*. Notably, CTHRC1 has recently been reported to promote invasion and metastasis of ovarian carcinoma cells in xenograft assays [83]. These experimental settings are, however, complicated by the fact that CTHRC1 is also expressed by stromal cells and thus for reliable results the experiments should be performed in CTHRC1-knockout animals. Further, the subcutaneous xenograft transplantation model does not recapitulate human melanomas, as it does not provide the proper microenvironment for the co-evolution of tumor and stromal cells taking place in actual human tumors. In any case, our studies on clinical human melanomas show that CTHRC1 expression in primary melanomas correlates with lymph node metastasis and poor patient prognosis. Further, we have found CTHRC1 to be expressed in lymph node macrometastases at a similar high level to that in primary melanomas ([35] and data not shown).

In conclusion, we found that several genes associated with inflammation, angiogenesis, and ECM modification were commonly upregulated during the development, progression, and metastasis of primary melanomas. Our results indicate that changes in the stromal gene expression and the tumor microenvironment promote tumor progression and metastasis, providing many new potential targets for therapy and markers to predict disease outcomes. These findings are consistent with recent studies on colorectal cancer [84] and other cancers (reviewed in [19, 85]). Here, we found that one such potential target gene, *CTHRC1*, was overexpressed by both melanoma cells and the surrounding activated fibroblasts, and showed high expression in tumor blood vessels as well. Interestingly, in melanoma cells, we found that CTHRC1 was coordinately expressed with the invasion- and angiogenesis-promoting genes *FN1*, *ITGB3*, and *NFATC2*. In functional analyses, we found that CTHRC1 was required for cell migration/invasion and may regulate the switch between the proliferative and invasive phenotypes. The expression of CTHRC1 was found to be induced by NFATC2, which has been implicated in the pathogenesis of many solid tumors, including pancreatic, lung, and breast cancers [86, 87]. NFATs may function as integrators of various oncogenic signaling pathways, including MAPK, Wnt, and Notch [88] and, thus, represent promising targets for cancer therapy. Targeting NFAT signaling specifically in the tumor cells and in the tumor endothelium may most effectively inhibit NFAT-promoted tumor progression [87]. It is notable that *CTHRC1* and many of the genes that we found to be associated with CTHRC1 overexpression during melanoma progression are also induced by TGFβ. Of further interest, the switch of the tumor-suppressive function of TGFβ towards tumor progression [89] has recently been reported to be driven by the activation of NFATs [90], and NFAT has been found to cooperate with TGFβ in inducing the epithelial—mesenchymal transition in breast cancer cells [91]. Thus, combined inhibition of both NFAT and TGFβ signaling pathways in melanomas represents an interesting possibility, since it may interfere with both tumor cell invasion and angiogenesis.

## MATERIALS AND METHODS

### Patient samples

We obtained fresh primary cutaneous melanomas (*n* = 36), melanoma lymph node metastases (*n* = 19), and benign nevi (*n* = 31) from healthy volunteers by surgical excision at Helsinki University Central Hospital using protocols approved by the Ethics Committee of Helsinki University Central Hospital. All patients provided informed consent. Half of each tissue specimen was fixed in formalin for histopathological and immunohistochemical analyses, and the other half was immediately frozen in liquid nitrogen or immersed in RNAlater RNA stabilization solution (Life Technologies, Carlsbad, CA, USA) for gene expression analyses using DNA microarrays and RT-PCR.

### RNA isolation and purification

RNA was extracted from cells and tissues using the RNeasy kit (>200 nt RNA, Qiagen, Hilden, Germany) or the *mir*Vana miRNA isolation kit (total-RNA, Life Technologies). Frozen tissue specimens were ground in liquid nitrogen and homogenized in lysis buffer with a 21-gauge needle. Tissues immersed in RNAlater were homogenized in lysis buffer with Lysing matrix D and the FastPrep FP120 Cell Disrupter (Qbiogene/MP Biomedicals, Santa Ana, CA, USA) according to the manufacturer's instructions. Pigment removal was performed by adsorption to Bio-Gel P-60 as described elsewhere [46]. The quality of the purified RNA was assessed by agarose gel electrophoresis or by Bioanalyzer 2100 (Agilent Technologies, Santa Clara, CA, USA).

### Microarray analysis

Normal nevi (*n* = 11) and primary melanomas (*n* = 21), as well as RNA from normal melanocytes, nevus cells, primary melanoma cells, and melanoma cell lines, were analyzed using the Human Genome U133 Plus 2.0 array (Affymetrix, Santa Clara, CA, USA) as previously described [46]. The microarray data is deposited in the ArrayExpress database (https://www.ebi.ac.uk/arrayexpress/), accession no. E-MTAB-1862.

### Statistical analysis of microarray data

The microarray probe signals were preprocessed using the RMA algorithm (RMAExpress 1.1.0, http://rmaexpress.bmbolstad.com/). Gene probe sets with a mean difference <100 and a change of <1.5-fold between different sample groups were filtered off before rank ordering using SAM [92] (http://www-stat.stanford.edu/~tibs/SAM/). SAM was performed using the samr package in R (version 3.1.2) (http://www.r-project.org/) using 4000 random permutations. Delta was chosen so that the false discovery rate (FDR) was <1%.

Gene lists were subjected to hypergeometric testing for GO classes and KEGG pathways using the Chipster v3.4.1 software (http://chipster.csc.fi/). We considered GO classes and pathways with *P*-values <0.05 and at least two observed genes as significantly overrepresented. Hierarchical clustering of genes was performed with the Chipster software using Pearson's correlation as the distance metric and average linkage as the clustering method. Gene lists were also subjected to gene enrichment analysis using the Molecular Signature Database (MSigDB, v5.0, Broad institute, MA, USA, http://www.broadinstitute.org/gsea/msigdb/index.jsp), Hallmark gene set collection.

### RT-PCR analyses and DNA sequencing

One μg of RNA was reverse-transcribed into cDNA as previously described [46] and used for the analysis of mRNA expression levels using semiquantitative and quantitative PCR. Semiquantitative RT-PCR analyses were performed as previously described [46], with the primers and PCR variables listed in Supplementary Table S8. The sequencing of PCR fragments is described elsewhere [35].

Real-time qRT-PCR analyses were performed using the ABI PRISM 7700 Sequence Detection System instrument and software (Life Technologies). Relative mRNA expression levels of CTHRC1 were measured using TaqMan assay Hs00298917_m1, that of AKR1C3 using Hs00366267_m1, WNT5A using Hs00998537_m1, and FABP7 using Hs00361426_m1. Expression levels of all genes were normalized with the pre-developed TaqMan assay for ribosomal protein, large, P0 (RPLP0) (Life Technologies).

### Immunohistochemistry

Formalin-fixed paraffin-embedded sections (5 μm) were deparaffinized, rehydrated in a graded ethanol series, and treated with trypsin (0.1% in PBS) at 37°C for 20 minutes (CTHRC1 and vWF) or subjected to heat-induced epitope retrieval in citrate buffer (0.01M, pH 6.0) (MCM7 and cleaved caspase 3) or in Tris-EDTA buffer (pH 8.0) (S100). The endogenous peroxidase activity was blocked with 0.3% H_2_O_2_ in methanol for 30 minutes. Sections were blocked with CAS-block reagent (Zymed Laboratories/Life Technologies) and/or 1.5% to 10% normal goat serum (Vector Laboratories, Burlingame, CA, USA) in PBS for 1 hour. A panel of CTHRC1 antibodies (N2C3 from Genetex, Irvine, CA, USA; 11647-RP02 from Sino Biological Inc., Beijing, China; ab85739 and ab54181 from Abcam, Cambridge, UK; clone 1G12 from Abnova, Taipei, Taiwan; clone Vli55 from Maine Medical Center Research Institute, Scarborough, ME, USA; H-213 from Santa Cruz Biotechnology, Dallas, TX, USA) were tested for their performance in immunohistochemistry, selecting the best for further analyses (Figures 3J, 3L, 3M, 3P and Supplementary Figure S9A). Specimens were then stained with the rabbit polyclonal antibodies (pAb) to CTHRC1 (N2C3, GeneTex; 1:200) and to vWF (A0082, DAKO/Agilent Technologies; 1:1000; for the visualization of blood vessels), or rabbit monoclonal antibodies to MCM7 (clone EP1974Y, Epitomics/Abcam; 1:100) or to cleaved caspase 3 (clone 5A1E, Cell Signaling Technology, Danvers, MA, USA; 1:200) in PBS containing 0.1% normal goat serum at 4°C overnight. For S100 staining, specimens were incubated with a rabbit pAb to S100 (Z-0311, Dako/Agilent Technologies; 1:100) for 30 minutes and detected using the ultraView Universal DAB Detection kit (Ventana/Roche) according to standard protocol.

Cryosections (5 μm) for CTHRC1 staining were fixed with 4% paraformaldehyde for 25 minutes and permeabilized in 0.1% Triton X-100 in PBS for 10 minutes. The endogenous peroxidase activity was blocked with 0.3% H_2_O_2_ in PBS for 10 minutes. Sections were blocked in 5% bovine serum albumin (BSA) and 10% normal goat serum in PBS for 1 hour, and incubated with rabbit pAb to CTHRC1 (11647-RP02, Sino Biological Inc.; 1:780) in PBS containing 5% normal goat serum at 4°C overnight. In CD31 and cFN staining, endogenous peroxidases were inactivated by 0.3% H_2_O_2_ in methanol for 30 minutes. Sections were blocked in 1% BSA and in CAS-block for 30 minutes (for CD31) or in 1% normal goat serum in PBS for 1 hour (for cFN), and incubated with the mouse monoclonal antibody (mAb) to CD31 (clone JC70A, DAKO/Agilent Technologies; 1:200) in CAS-block or cFN (clone FN-3E2, Sigma-Aldrich, St. Louis, MO, USA; 1:400) in PBS containing 1% normal goat serum at 4°C overnight. In all stainings, normal mouse or rabbit IgG (of the same isotype when applicable) served as the negative control (shown for CTHRC1 in Figure 3M, 3P, and in Supplementary Figure S9A). Immunodetection for both the paraffin-embedded and cryosections was performed using the Vectastain ABC kit (Vector Laboratories) according to the manufacturer's protocol using 3-amino-9-ethylcarbatzole (AEC) or 3, 3 —diaminobenzidine (DAB) as the chromogen. Sections were counterstained with Mayer's hematoxylin and mounted with Aquamount. Images were taken using a Nikon Eclipse 80i microscope, a Digital Sight DS-5M camera, and the NIS-Elements F 2.20 software (Nikon, Tokyo, Japan).

### Western blotting

We performed the analysis of proteins from whole-cell lysates [93] and conditioned media [94, 95] as previously described. A panel of CTHRC1 antibodies (N2C3 from Genetex; 11647-RP02 from Sino Biological Inc.; ab85739, ab54181, ab135716, and clone 16D04 from Abcam; clone 1G12 from Abnova; H-213 from Santa Cruz Biotechnology) were tested for their performance in Western blotting, and the rabbit pAbs ab85739 and 11647-RP02 (for immunoblotting under nonreducing conditions) were selected for the study. The specificity of the antibodies was tested by analyzing the CTHRC1 protein in the control and CTHRC1-knockdown cells using Western blotting and immunocytochemistry (Supplementary Figure S5B, 5C, 5E, 5G, and Supplementary Figure S9B-9D). In addition, rabbit pAb to ITGB3 (H-96, Santa Cruz Biotechnology), rabbit mAb to phospho-Erk1/2 (20G11, Cell Signaling Technology), rabbit antiserum to cleaved caspase 3 (anti-hCCP32-p17, Merck Frosst, Quebec, Canada), and mouse mAbs to FN1 (NCL-FIB, Novocastra/Leica Biosystems, Wetzlar, Germany) and NFATC2 (BD Biosciences, Franklin Lakes, NJ, USA or 4G6-G5, Santa Cruz Biotechnology) were used to detect the respective proteins. Mouse mAbs to alpha-tubulin (DM1A, Abcam) and actin (JLA20, Merck Millipore, Billerica, MA, USA) were used as the loading controls. The densities of the protein bands were quantified using Image Studio Lite (LI-COR Biotechnology, Lincoln, NE, USA).

### *N*-glycosidase F-treatment

Proteins from cell lysates (50 μg) and conditioned medium (30 μg) were treated (after reduction and denaturation) with or without recombinant *N*-glycosidase F (Roche, Mannheim, Germany) (0.6 units/μg of protein) in sodium phosphate buffer (100 mmol/L, pH 7.2) supplemented with 1% NP-40 and 1X Complete protease inhibitor (Roche) at 37°C overnight.

### Cell culture

We isolated and cultured primary human melanocytes and melanoma cells as described elsewhere [96]. The human VGP melanoma cell lines WM115 (BRAF V600D mutation; from ATCC-LGC Standards, Borås, Sweden) and WM793 (BRAF V600E; provided by Dr. Meenhard Herlyn, Wistar Institute, Philadelphia, PA, USA), the metastatic melanoma cell lines MM170 (BRAF V600E; from CellBank Australia, Westmead, Australia), SKMEL-28 (BRAF V600E; from ATCC-LCG), and WM239 (BRAF V600D; from Dr. M. Herlyn), as well as the primary human adult and embryonic skin fibroblasts (provided by Drs. A.-M. Ranki and A. Vaheri, University of Helsinki, Finland, respectively) were cultured in RPMI 1640 medium (Sigma-Aldrich) supplemented with 10% FBS (Gibco/Life Technologies) and antibiotics. Metastatic melanoma cell lines SKMEL-103 (NRAS Q61R) and SKMEL-147 (NRAS Q61R) (both originating from Dr. Alan Houghton, Memorial Sloan-Kettering Cancer Center, New York, NY, USA; provided by Dr. Maria Soengas, Spanish National Cancer Research Center, Madrid, Spain) as well as the BLM cell line (NRAS Q61R; from Dr. van Muijen, Radboud University, Nijmegen Medical Centre, Nijmegen, Netherlands) were cultured in Dulbecco's Modified Eagle Medium (Gibco/Life Technologies) supplemented with 10% FBS and antibiotics. Primary human microvascular endothelial cells (Life Technologies) were cultured in growth factor—supplemented Medium 131 (Gibco/Life Technologies).

### Inhibitors and blocking antibodies of signaling pathways

CsA was purchased from Santa Cruz Biotechnology, the TGFβ receptor I (ALK5) inhibitor SB505124 from Sigma-Aldrich, and the BRAF inhibitor Dabrafenib (GSK2118436A) from Selleckchem (Houston, TX, USA). The function-blocking antibody to integrin αvβ3 (LM609) was acquired from Merck Millipore.

### Short hairpin RNA lentiviral particle transduction

WM239, SKMEL-28, and MM170 were transduced with shRNA lentiviral particles (Santa Cruz Biotechnology) targeting CTHRC1 (sc-77043-V) or with scrambled control shRNA particles (sc-108080) in duplicate in two separate experiments according to the manufacturer's instructions. Pools of puromycin-resistant cells were used in the assays.

### Transfection of small interfering RNA

WM239 cells were transfected with siRNA duplexes targeting the full-length CTHRC1 (sense strand: 5′-GCCAGACGCUGACCACGUUCCUCdTdC-3′; antisense strand: 5′-GAGAGGAACGUGGUCAGCGUCUGGCUC-3′;), the variant 3 CTHRC1 (sense strand: 5′-CAGGUAGGAGCAUCACAGUCAAGdCdT-3′; antisense strand: 5′-AGCUUGACUGUGAUGCUCCUACCUGGC-3′; both designed using the Integrated DNA Technologies RNAi Design Tool and manufactured by Integrated DNA Technologies, Coralville, IA, USA), or with duplexes targeting all CTHRC1 variants (sc-77043) or with scrambled control siRNA duplexes (sc-37007, both from Santa Cruz Biotechnology) according to Santa Cruz Biotechnology's instructions. The cells were used in assays 48 hours after transfection. In addition, WM239 cells were transfected with duplexes targeting NFATC2 (sc-36055, Santa Cruz Biotechnology).

### Immunofluorescence staining

Cells were grown on glass coverslips for two to three days and fixed with 4% paraformaldehyde for 30 min. After permeabilization with 0.1% Triton X-100 in PBS for 10 min and blocking with 1% BSA (Invitrogen/Life Technologies) and 10% normal goat serum in PBS for 1 hour, the cells were incubated with 5 μg/ml of rabbit pAb to CTHRC1 (11647-RP02, Sino Biological Inc.) diluted in PBS containing 5% normal goat serum for 2 hours. Then, cells were washed three times with PBS and incubated with Alexa Fluor 488-conjugated goat anti-rabbit secondary antibodies (Invitrogen/Life Technologies) in PBS containing 1% normal goat serum. F-actin was immunostained with Alexa Fluor 594-conjugated phalloidin (Molecular Probes/Life Technologies). Finally, the cells were mounted with the Vectashield H-1200 mounting media containing DAPI (Vector Laboratories), and images were obtained using a Zeiss Axiophot2 epifluorescence microscope (Carl Zeiss, Oberkochen, Germany), QImaging Retiga 4000R digital camera, and QCapture Pro 6-software (QImaging, Surrey, Canada).

### Cell adhesion assay

Flat-bottomed 96-well plates were coated with 100 μl of 10 μg/ml BSA (fatty acid-free, Sigma-Aldrich), cFN (USBiologicals, Salem, MA), COL-I (BD Biosciences), and recombinant human CTHRC1 (Sino Biological Inc.) for 2 hours at 37°C, and washed three times with PBS. Cells (2 × 10^4^) were added to the wells in serum-free medium, and attached and spread cells were photographed and counted after incubation for 1 hour at 37°C.

### Cell migration assay

Cells (3 × 10^4^) were added to a Falcon cell culture insert (8 μm, BD Biosciences) in serum-free medium (200 μl), and the lower chamber was filled with 800 μl of growth medium. After incubation for 22 or 42 hours, the cells were fixed in 3.5% paraformaldehyde and stained with 0.5% crystal violet (in 20% methanol). Nonmigrated cells on the upper surface were scraped off, and migrated cells on the lower surface were photographed and counted. Experiments were repeated three times, and the results were analyzed using a two-tailed *t*-test.

### Three-dimensional collagen I gel and matrigel invasion assays

Melanoma cell invasion was assayed in a 3D collagen I gel (COL-I, high concentration, rat tail) and growth factor—reduced Matrigel (both from BD Biosciences) as described previously [34, 94]. Briefly, 30,000 WM239, SKMEL-28, or MM170 cells were cast between two gel layers, and medium supplemented with 10% serum was added on top of the gels (replenished every third day). The invasion patterns of the cells were analyzed daily using microscopy and photography.

### Xenograft nude mice assays

Animal studies were carried out according to the Animal Experiment Board in Finland (ELLA) for the care and use of animals under the licenses ESAVI-6285-04.10.07-20151. CTHRC1 shRNA- and control shRNA-expressing WM239 cells were injected (6 × 10^6^ cells in 75 μl of Opti-MEM I [Gibco/Life Technologies] with 75 μl of growth factor-reduced Matrigel or in 100 μl of Opti-MEM I) subcutaneously into the lower flank of 5 and 7-week-old female BALB/c nu mice in the two experiments, six animals per group (one inoculation per animal). Tumor growth was followed two to three times per week using a caliper (measuring in three dimensions), and the mice were euthanized at day 36 when the largest tumors reached the ethical limits. The tumors were removed, cut in half, and one half was fixed in formalin and embedded in paraffin and the other half was frozen in isopentane. For histological and immunohistochemical analyses, 5-μm sections were cut from paraffin blocks and stained with H&E and with antibodies against MCM7, active caspase 3, and S100, as described in the Immunohistochemistry section above. Part of the frozen tissue was subjected to RNA extraction and qRT-PCR for CTHRC1 expression analysis, as described in the RT-PCR analyses section.

### Statistical analysis

Statistical analyses were performed using a two-tailed Welch's *t*-test, where we considered *P* <0.05 significant. Survival curves were plotted according to the Kaplan-Meier method and compared using the log-rank test in the SPSS 21.0 software program (SPSS, Chicago, USA).

## SUPPLEMENTARY FIGURES AND TABLES











## Abbreviations

BSA: bovine serum albumin
cFN: cellular fibronectin
COL-I: collagen I
CsA: cyclosporine A
ECM: extracellular matrix
FDR: false discovery rate
GO: Gene Ontology
mAb: monoclonal antibody
pAb: polyclonal antibody
qRT-PCR: quantitative RT-PCR
SAM: Significance Analysis of Microarrays
shRNA: short hairpin RNA
siRNA: small interfering RNA
VGP: vertical growth phase
