# Supplementary material for: Gene expression analyses of primary melanomas reveal CTHRC1 as an important player in melanoma progression
Source: Oncotarget. 2016 Feb 23;7(12):15065–92. doi: 10.18632/oncotarget.7604 (PMC4924771; doi:10.18632/oncotarget.7604)
Supplement: Supplementary file 1 [file oncotarget-07-15065-s001.pdf]

SUPPLEMENTARY FIGURES AND TABLES

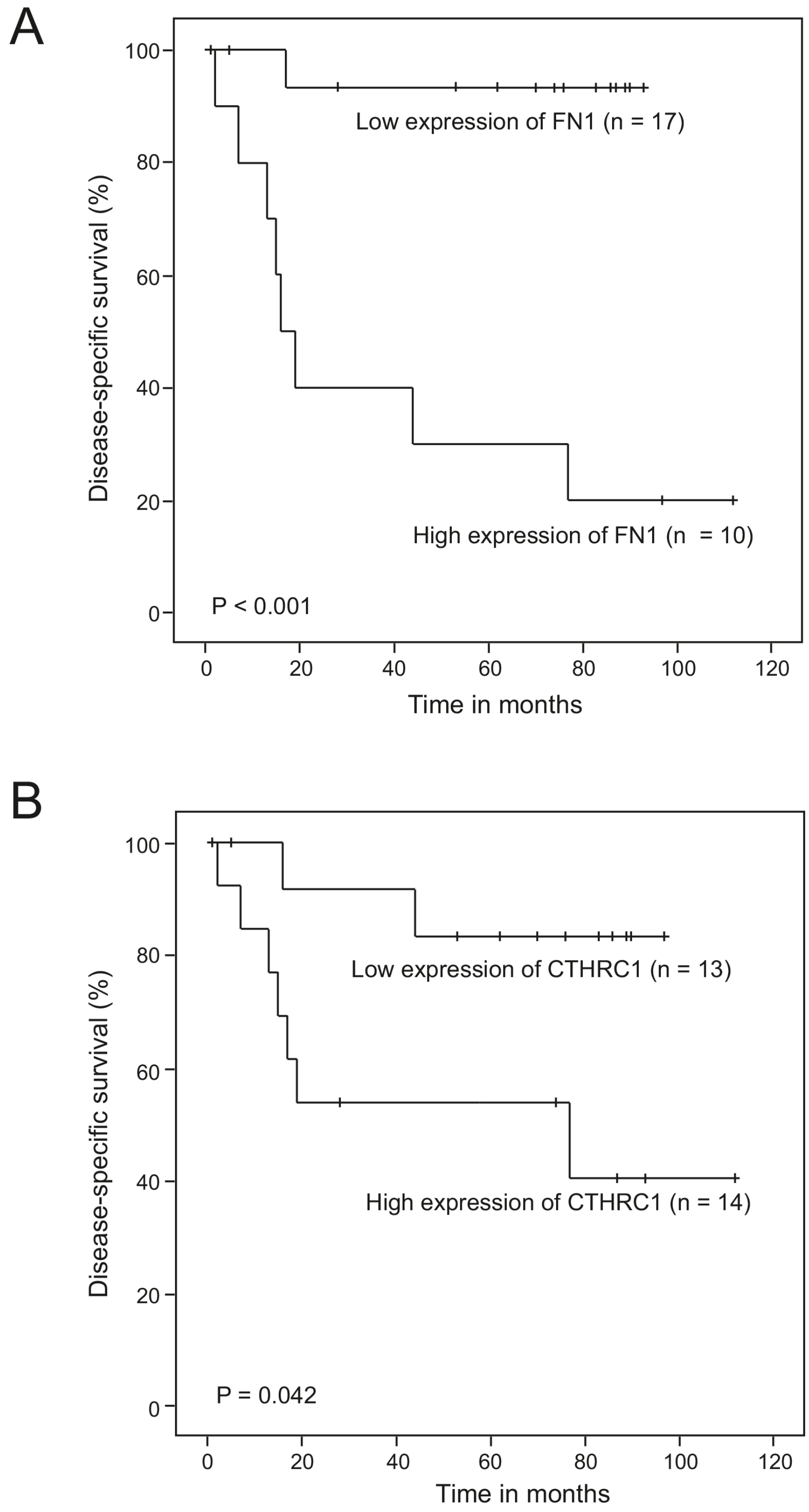

Supplementary Figure S1: The prognostic values of FN1 and CTHRC1 expression in primary melanomas. A–B. Kaplan-Meier survival curves for patient groups with primary melanomas showing low and high FN1 (A) and CTHRC1 (B) mRNA expression.

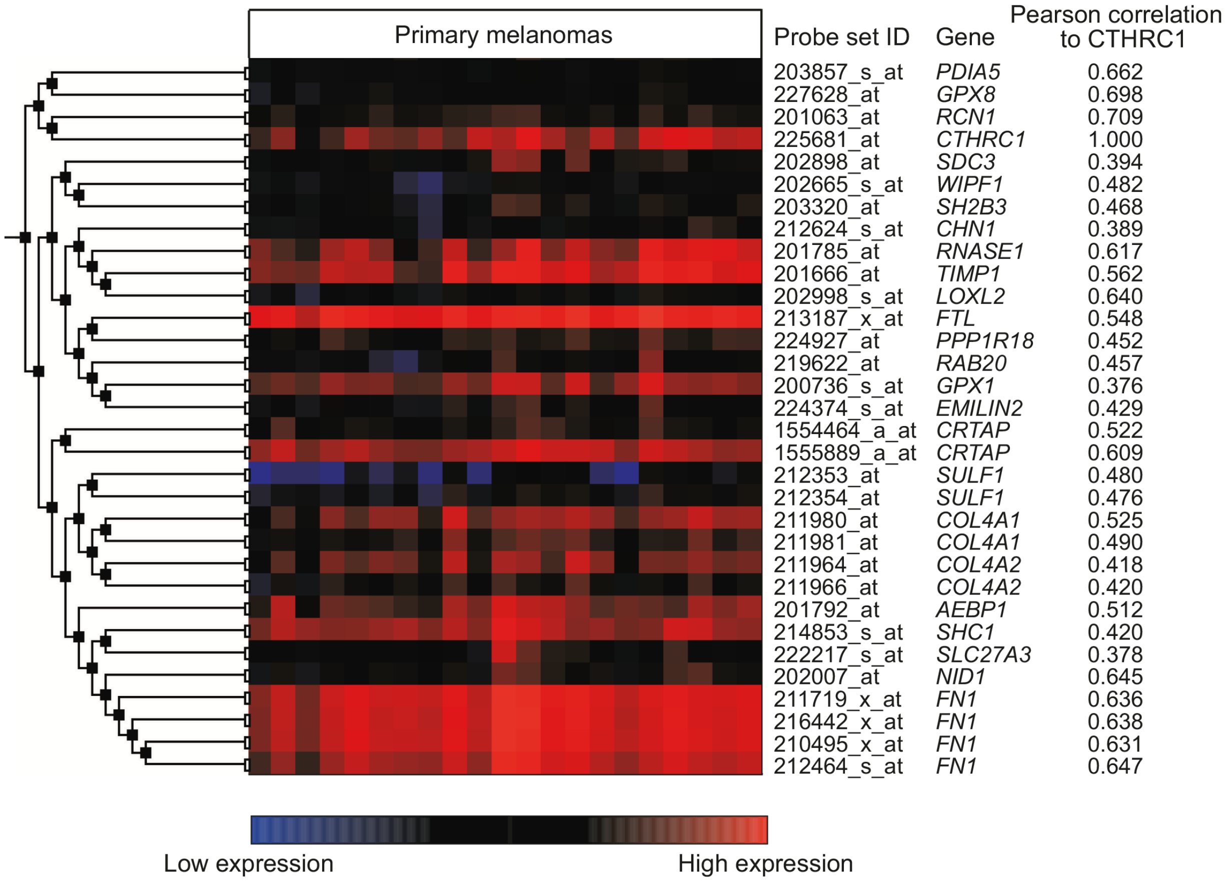

Supplementary Figure S2: Genes expressed coordinately with CTHRC1 in primary melanomas. Genes with a <100 mean difference and a <1.5 fold-change between benign nevi and primary melanomas were filtered off. The remaining 2981 probe sets were analyzed by hierarchical gene clustering in 21 primary melanomas.

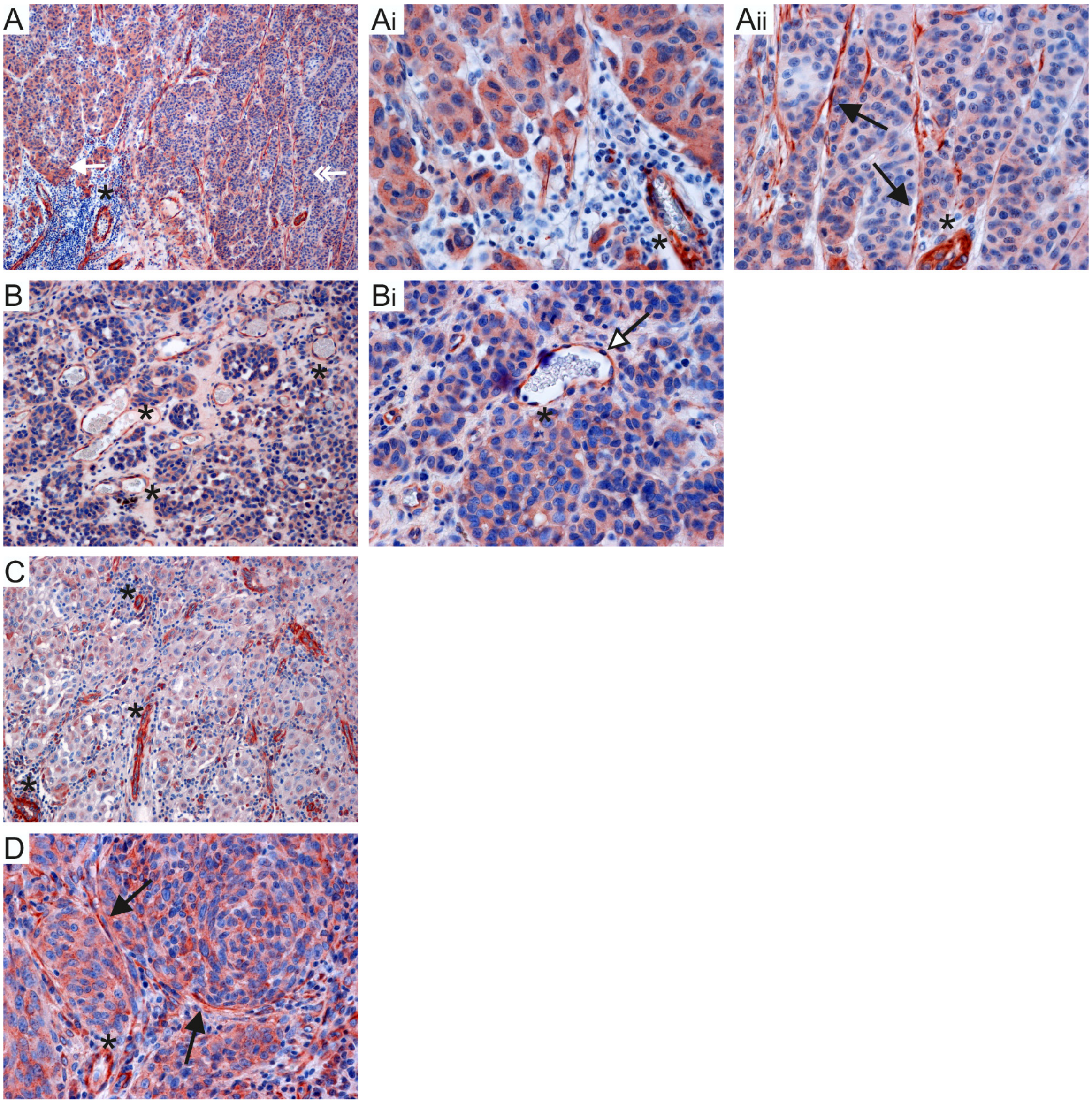

Supplementary Figure S3: Immunohistochemical staining of CTHRC1 in primary melanomas. A–D. Primary melanomas showing CTHRC1 staining in melanoma cells, fibroblasts, and/or blood vessels. Panels **Ai.** and **Aii.** are magnifications of the areas marked with a white arrow and a white double-headed arrow in panel (A), respectively. Panel **Bi.** shows a magnification of a blood vessel from a primary melanoma presented in panel **B.** Note the CTHRC1 staining in endothelial cells in (Bi); marked with a white-headed arrow. Examples of fibroblasts are marked with black arrows and blood vessels with asterisks. Original magnification 100x (A), 200x (B, C), 400x (Ai, Aii, Bi, D).

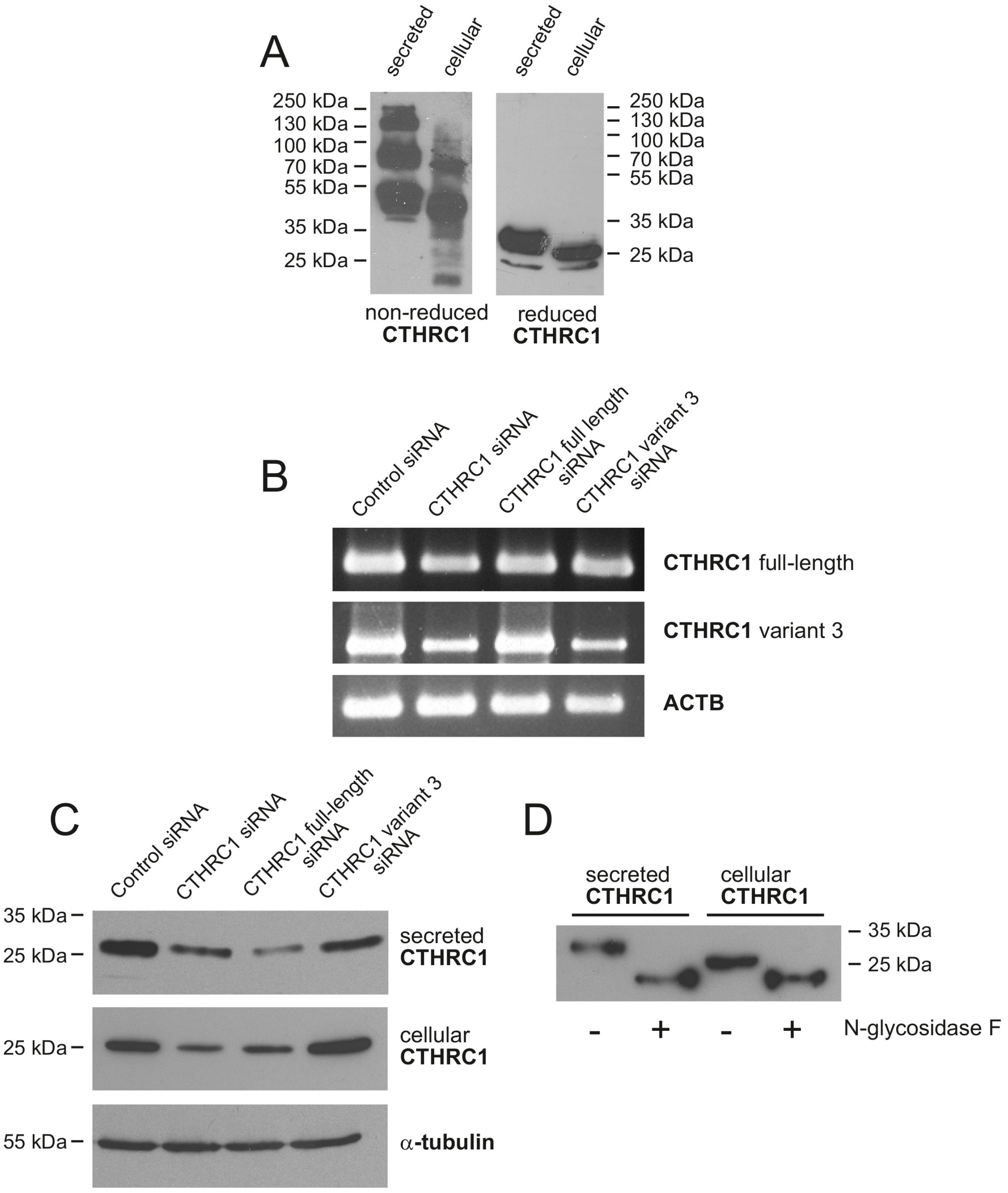

Supplementary Figure S4: Characterization of the secreted and cellular CTHRC1 protein. A. Western blot analysis of CTHRC1 in the conditioned media and cellular lysate of WM239 melanoma cells under non-reducing and reducing conditions. **B.** Expression of full-length and variant CTHRC1 mRNA in WM239 control and CTHRC1-knockdown cells as analyzed by semiquantitative RT-PCR. Actin (ACTB) was used as a control. **C.** Western blot analysis of CTHRC1 in the conditioned media and cellular lysates of WM239 control and CTHRC1-knockdown cells. Alpha-tubulin was used as a loading control. **D.** Western blot analysis of CTHRC1 in the conditioned media and cellular lysate of WM239 cells with and without N-glycosidase F-treatment.

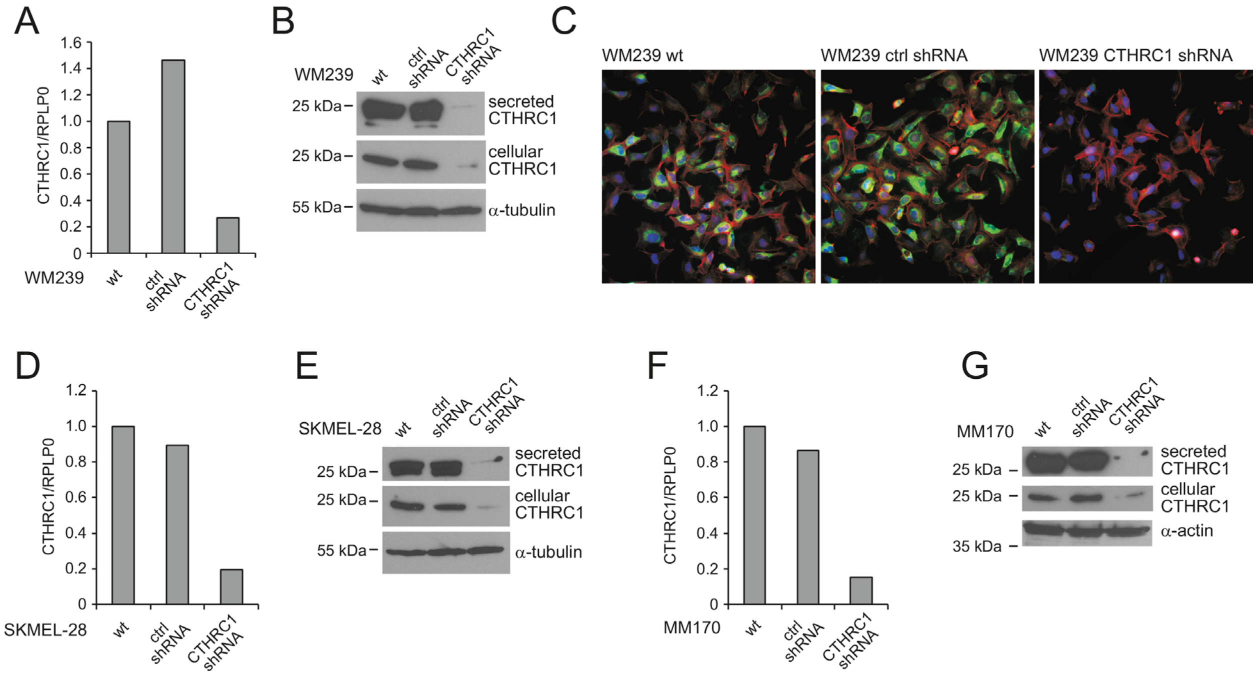

Supplementary Figure S5: CTHRC1 down-regulation in CTHRC1-knockdown cells. Relative CTHRC1 mRNA levels in WM239 **A.** SKMEL-28 **D.** and MM170 **F.** wild-type (wt) cells and cells transduced with lentiviral control (ctrl) shRNAs or shRNAs targeting CTHRC1. CTHRC1 and RPLP0 cDNA levels were measured by qRT-PCR in triplicate for each sample. Western blot analysis of CTHRC1 in the conditioned media and cellular lysates of WM239 **B.** SKMEL-28 **E.** and MM170 **G.** wild-type cells and cells transduced with lentiviral control shRNAs or shRNAs targeting CTHRC1. Alpha-tubulin or alpha-actin was used as a loading control. **C.** Immunofluorescence staining of CTHRC1 in WM239 wild-type, control shRNA, and CTHRC1-knockdown cells. CTHRC1 is seen in green, F-actin in red, and the nuclei (DAPI) in blue. Original magnification x200.

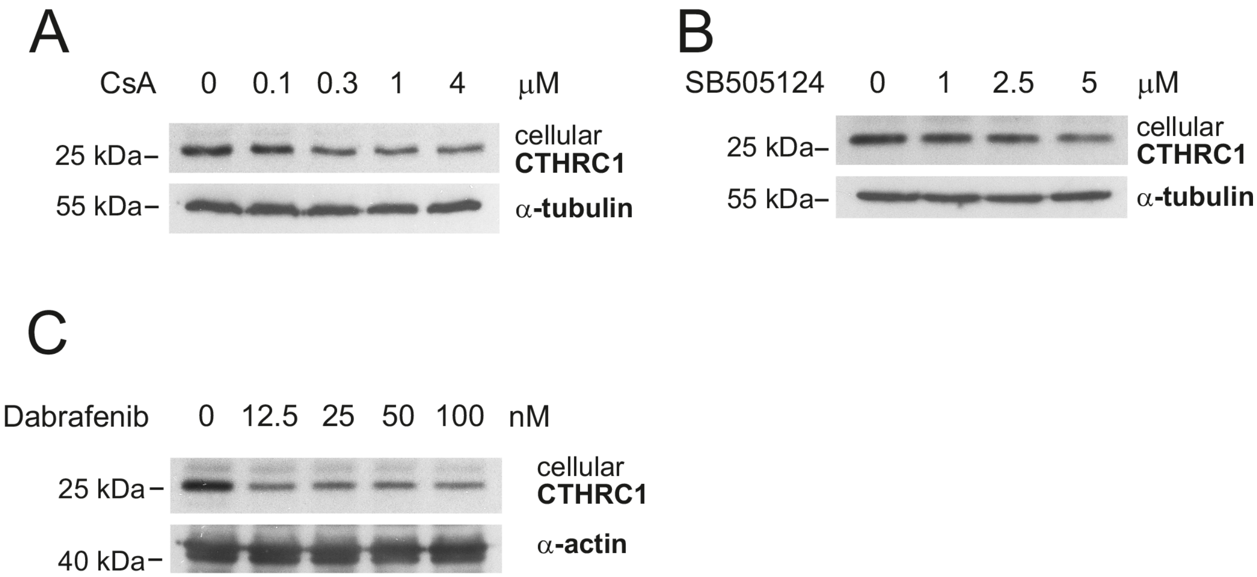

Supplementary Figure S6: Regulation of CTHRC1 expression. A. Western blot analysis of CTHRC1 in MM170 cells treated without or with increasing concentrations of cyclosporin A (CsA) for 24 hours (A) **B–C.** TGFβ-receptor I/ALK-5 inhibitor SB505124 for 48 hours (B) and BRAF inhibitor Dabrafenib for 24 hours (C). Alpha-tubulin or alpha-actin were used as a loading control.

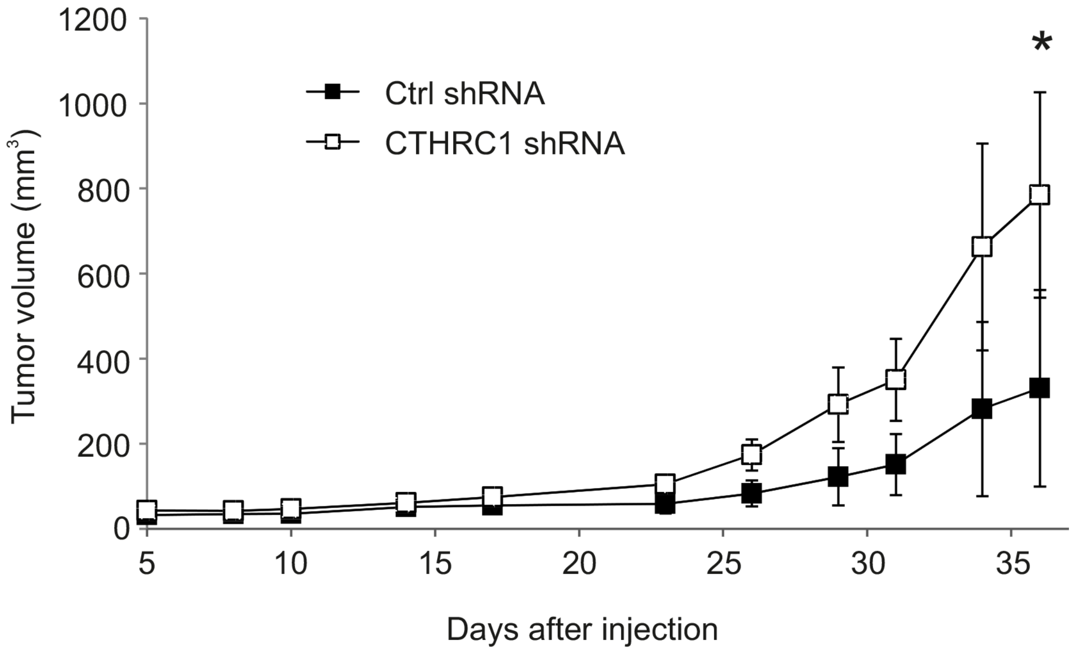

Supplementary Figure S7: Growth curves of tumors induced by control (ctrl) shRNA-expressing and CTHRC1-knockdown WM239 cells in nude mice. Cells (6 x 106) were mixed with Matrigel and injected subcutaneously into the lower flank of the individual mice (six mice per group). Tumor volume was measured with a caliper two to three times per week. Bars represent standard deviations. *P < 0.01.

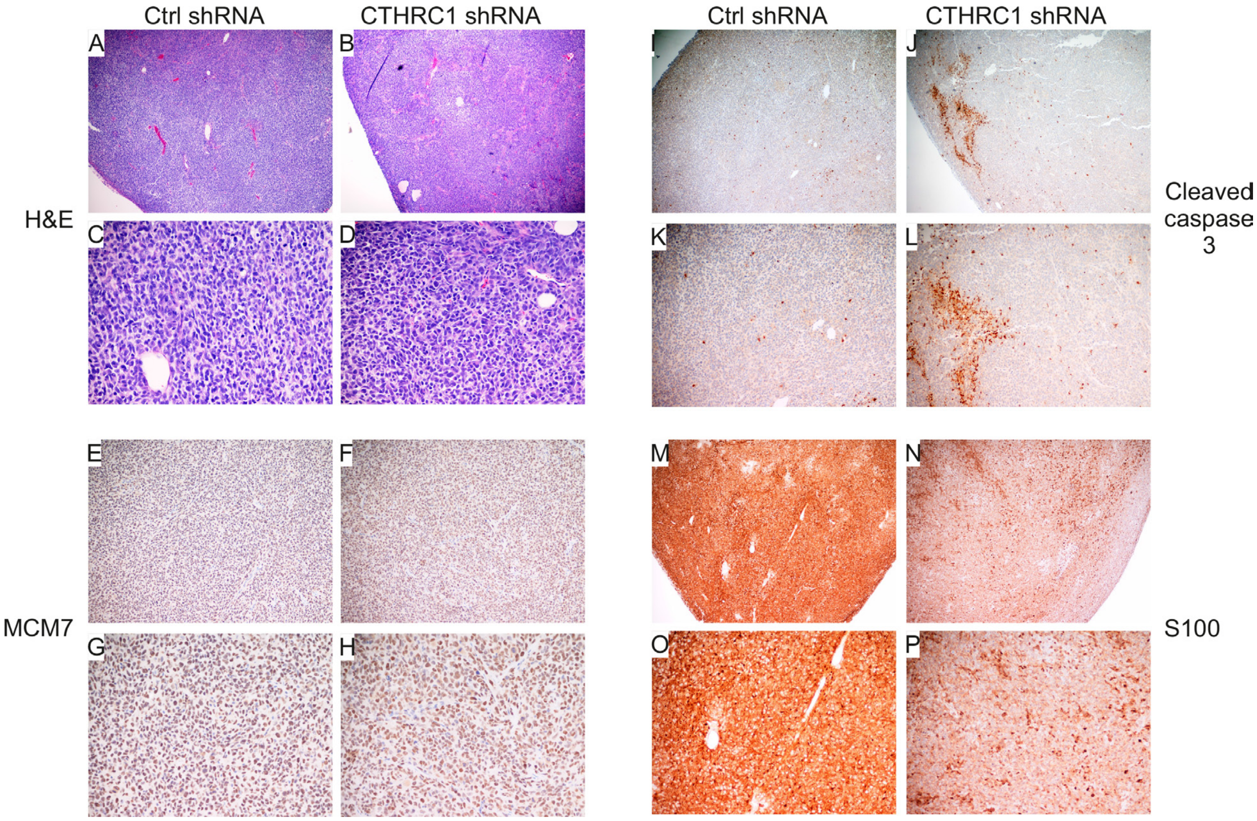

Supplementary Figure S8: Histochemical analysis of tumors induced by control (ctrl) shRNA-expressing and CTHRC1-knockdown WM239 cells in nude mice. A–D. Representative H&E stainings of control (A and C) and CTHRC1-knockdown tumors (B and D). **E-P.** Representative immunohistochemical stainings of MCM7 in control (E and G) and CTHRC1-knockdown tumors (F and H), cleaved caspase 3 in control (I and K) and CTHRC1-knockdown tumors (J and L), and S100 in control (M and O) and CTHRC1-knockdown tumors (N and P). (E-P) Positive immunostaining is shown in brown (DAB). Original magnification 40x (A, B, I, J, M, N), 100x (E, F, K, L, O, P), 200x (C, D, G, H).

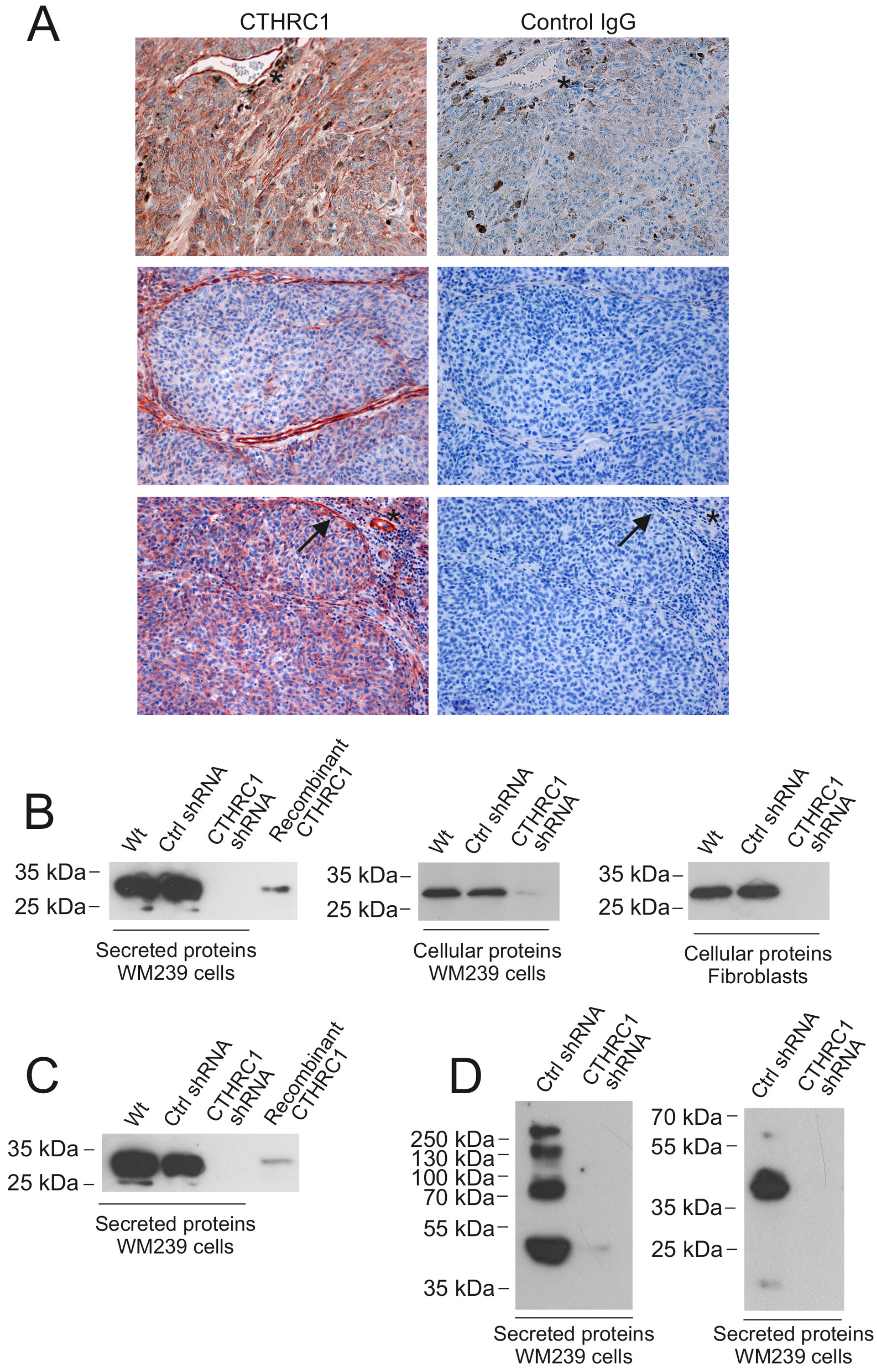

Supplementary Figure S9: Specificity of the CTHRC1 antibodies in immunohistochemical and western blotting analyses. **A.** Consecutive sections from the same primary melanomas stained with the Genetex antibody recognizing CTHRC1 and with a normal rabbit IgG control antibody. Positive immunostaining is shown in red (AEC). Original magnification 200x. Examples of fibroblasts are marked with arrows and blood vessels with asterisks. **B–D.** Western blot analysis of recombinant CTHRC1 protein as well as CTHRC1 in the conditioned media and/or cellular lysates of wild-type (wt), control (ctrl) and CTHRC1-knockdown WM239 melanoma cells and/or fibroblasts using the rabbit polyclonal antibodies from Genetex (N2C3) (B) Abcam (ab85739) (C) and Sino Biological (11647-RP02; non-reduced) (D).

Supplementary Table S1: Overexpressed genes (≥ 4-fold shown in bold) in primary melanomas compared to benign nevi by Significance Analysis of Microarrays (SAM)

See Supplementary File 1

Supplementary Table S2: Underexpressed genes in primary melanomas compared to benign nevi by Significance Analysis of Microarrays (SAM)

See Supplementary File 2

Supplementary Table S3: Overexpressed genes in metastatic compared to non-metastatic primary melanomas by Significance Analysis of Microarrays (SAM)

See Supplementary File 3

Supplementary Table S4: Underexpressed genes in metastatic compared to non-metastatic primary melanomas by Significance Analysis of Microarrays (SAM)

See Supplementary File 4

Supplementary Table S5: Genes correlating with CTHRC1 expression both in our panel of primary cells and cell lines as well as in a panel of 34 melanoma cell lines obtained from a data bank

Gene	Gene description	Probe set ID	Pearson correlation	
			Primary cells and cell lines	Cell lines (E-GEOD-7152)
CTHRC1	Collagen triple helix repeat containing 1	225681_at	1.000	1.000
FN1	Fibronectin 1	211719_x_at	0.944	0.802
FN1	Fibronectin 1	216442_x_at	0.943	0.790
FN1	Fibronectin 1	210495_x_at	0.938	0.794
FN1	Fibronectin 1	212464_s_at	0.921	0.797
GPR126	G protein-coupled receptor 126	213094_at	0.881	0.655
FN1	Fibronectin 1	214701_s_at	0.854	0.658
NFATC2	Nuclear factor of activated T-cells, cytoplasmic, calcineurin-dependent 2	228442_at	0.822	0.662
NFATC2	Nuclear factor of activated T-cells, cytoplasmic, calcineurin-dependent 2	226991_at	0.803	0.645
ITGB3	Integrin, beta 3 (platelet glycoprotein IIIa, antigen CD61)	204627_s_at	0.785	0.604

Supplementary Table S6: Genes downregulated in WM239 melanoma cells after knockdown of CTHRC1

Gene	Gene description	Probe set ID	Signal WM239 cells		Fold
			Control shRNA	CTHRC1 shRNA	Control vs CTHRC1 shRNA
CTHRC1	Collagen triple helix repeat containing 1	225681_at	9375	3349	2.8
AKR1C3	Aldo-keto reductase family 1, member C3	209160_at	183	72	2.5
RGS2	Regulator of G-protein signaling 2, 24kDa	202388_at	1467	709	2.1
SLC7A11	Solute carrier family 7, member 11	217678_at	1050	513	2.0
GHITM	Growth hormone inducible transmembrane protein	209248_at	1222	619	2.0
TMF1	TATA element modulatory factor 1	214948_s_at	1341	703	1.9
ISCA1	Iron-sulfur cluster assembly 1 homolog	209274_s_at	587	322	1.8
SPANXC/ SPANXE	SPANX family, member C/ SPANX family, member E	220217_x_at	451	248	1.8
FABP7	Fatty acid binding protein 7, brain	205029_s_at	688	379	1.8
HS3ST3A1	Heparan sulfate (glucosamine) 3-O-sulfotransferase 3A1	219985_at	248	137	1.8
SPANXA1/A2/ B1/B2/C/E/F1	Sperm protein associated with the nucleus, X-linked, family member A1/ A2/B1/ B2/C/E/F1	220922_s_at	514	284	1.8
UG0898H09	Uncharacterized LOC643763	1558388_a_at	248	140	1.8
PRKCI	Protein kinase C, iota	213518_at	594	337	1.8
CISD2	CDGSH iron sulfur domain 2	226686_at	297	175	1.7
PREPL	Prolyl endopeptidase-like	212215_at	449	267	1.7
MYL12A	Myosin, light chain 12A, regulatory, non-sarcomeric	201319_at	1690	1017	1.7
TMX1	Thioredoxin-related transmembrane protein 1	208097_s_at	1289	789	1.6
WNT5A	Wingless-type MMTV integration site family, member 5A	205990_s_at	472	289	1.6
ARHGDIB	Rho GDP dissociation inhibitor (GDI) beta	201288_at	400	246	1.6

(*Continued*)

Gene	Gene description	Probe set ID	Signal WM239 cells		Fold
			Control shRNA	CTHRC1 shRNA	Control vs CTHRC1 shRNA
EXOC5	Exocyst complex component 5	228418_at	566	350	1.6
TMEM9B	TMEM9 domain family, member B	222507_s_at	432	269	1.6
SAT1	Spermidine/spermine N1-acetyltransferase 1	203455_s_at	410	256	1.6
TSPAN13	Tetraspanin 13	217979_at	3346	2100	1.6
KIAA1199	KIAA1199	212942_s_at	1291	817	1.6
RUNX2	Runt-related transcription factor 2	232231_at	342	218	1.6
SLC6A6	Solute carrier family 6 (neurotransmitter transporter, taurine), member 6	228754_at	1117	712	1.6
MGP	Matrix Gla protein	202291_s_at	498	317	1.6
MRGPRX3	MAS-related GPR, member X3	1553293_at	999	639	1.6
CFL1	Cofilin 1 (non-muscle)	1555730_a_at	816	527	1.5
CALD1	Caldesmon 1	201617_x_at	1234	806	1.5
IRS1	Insulin receptor substrate 1	204686_at	293	192	1.5
MESDC2	Mesoderm development candidate 2	224675_at	1246	820	1.5
SEPT11	Septin 11	201307_at	576	380	1.5
STIP1	Stress-induced-phosphoprotein 1	212009_s_at	537	356	1.5
C16orf95	Chromosome 16 open reading frame 95	219785_s_at	435	289	1.5
ENO1	Enolase 1, (alpha)	217294_s_at	4517	3000	1.5

Supplementary Table S7: Changes in mRNA expression levels of selected genes in control and CTHRC1-knockdown cells analyzed by qRT-PCR

Gene	Gene description	WM239[a]	WM239[b]	Fold control vs CTHRC1 shRNA	
				MM170	MM170[b]
CTHRC1	Collagen triple helix repeat containing 1	5.5	10.9	7.0	12.0
AKR1C3	Aldo-keto reductase family 1, member C3	2.5	6.7	6.2	5.5
FABP7	Fatty acid binding protein 7, brain	2.0	5.1	1.7	1.4
WNT5A	Wingless-type MMTV integration site family, member 5A	1.5	2.1	1.4	2.0

[a]WM239 control and CTHRC1-knockdown cells also analyzed by microarrays, see Supplementary Table S6.
[b] WM239/MM170 control and CTHRC1-knockdown cells from a separate transfection experiment.Relative expression levels of genes were normalized to that of RPLP0.

Supplementary Table S8: PCR variables

Gene	1)	Sequence 5' to 3'	2)	3)	Source
ACTB	F<br>R	GCTCGTCGTCGACAACGGCTC<br>CAAACATGATCTGGGTCATCTTCTC	55	20	Invitrogen/Life Technologies
CTHRC1 full-length	F<br>R	AGCGCCTCTGAGATCCCCAA<br>TGAACAAGTGCCAACCCAGA	59	24	Park et al. 2013
variant 3	F<br>R	AGAAGGTTTAAGGCCGGAAAGGGA<br>GTCATTTAAGTGAACCATTCCAAGGC	57	35	PrimerQuest
FN1 EDA domain	F<br>R	GGAGAGAGTCAGCCTCTGGTTCAG<br>TGTCCACTGGGCGCTCAGGCTTGTG	56	25	Palmieri et al. 1999
EDB domain	F<br>R	CGGCCTGGAGTACAATGTCAGTGT<br>CAGGTGACACGCATGGTGTCTGGA	56	25	Palmieri et al. 1999
ITGB3	F<br>R	GATGCATCCCACTTGCTGGTGTTT<br>CATTGTTGAGGCAGGTGGCATTGA	56	23	PrimerQuest
NFATC2	F<br>R	AAGCCACGGTGGATAAGGACAAGA<br>ACATGATGTGCTGGAACTCCTGGT	56	25	PrimerQuest

1) Primer orientation, F=forward, R=reverse; 2) Annealing temperature (°C); 3) Number of PCR cycles.

Park EH, Kim S, Jo JY, Kim SJ, Hwang Y, Kim JM, Song SY, Lee DK, Koh SS: Collagen triple helix repeat containing-1 promotes pancreatic cancer progression by regulating migration and adhesion of tumor cells. Carcinogenesis 2013, 34:694-702.

Palmieri G, Strazzullo M, Ascierto PA, Satriano SM, Daponte A, Castello G: Polymerase chain reaction-based detection of circulating melanoma cells as an effective marker of tumor progression. Melanoma Cooperative Group. J Clin Oncol 1999, 17:304-311.
